# Characterization of fossilized relatives of the White Spot Syndrome Virus in genomes of decapod crustaceans

**DOI:** 10.1186/s12862-015-0380-7

**Published:** 2015-07-19

**Authors:** Andrey Rozenberg, Philipp Brand, Nicole Rivera, Florian Leese, Christoph D. Schubart

**Affiliations:** Ruhr University Bochum, Department of Animal Ecology, Evolution and Biodiversity, Bochum, Germany; University of Regensburg, Department of Zoology and Evolutionary Biology, Regensburg, Germany; University of California, Davis, Department of Evolution and Ecology, Center for Population Biology, Davis, USA

**Keywords:** Nimaviridae, WSSV, White Spot Syndrome Virus, Endogenized viruses, Paleovirology

## Abstract

**Background:**

The White Spot Syndrome Virus (WSSV) is an important pathogen that infects a variety of decapod species and causes a highly contagious disease in penaeid shrimps. Mass mortalities caused by WSSV have pronounced commercial impact on shrimp aquaculture. Until now WSSV is the only known member of the virus family Nimaviridae, a group with obscure phylogenetic affinities. Its isolated position makes WSSV studies challenging due to large number of genes without homology in other viruses or cellular organisms.

**Results:**

Here we report the discovery of an unusually large amount of sequences with high similarity to WSSV in a genomic library from the Jamaican bromeliad crab *Metopaulias depressus. De novo* assembly of these sequences allowed for the partial reconstruction of the genome of this endogenized virus with total length of 200 kbp encompassed in three scaffolds. The genome includes at least 68 putative open reading frames with homology in WSSV, most of which are intact. Among these, twelve orthologs of WSSV genes coding for non-structural proteins and nine genes known to code for the major components of the WSSV virion were discovered. Together with reanalysis of two similar cases of WSSV-like sequences in penaeid shrimp genomic libraries, our data allowed comparison of gene composition and gene order between different lineages related to WSSV. Furthermore, screening of published sequence databases revealed sequences with highest similarity to WSSV and the newly described virus in genomic libraries of at least three further decapod species. Analysis of the viral sequences detected in decapods suggests that they are less a result of contemporary WSSV infection, but rather originate from ancestral infection events. Phylogenetic analyses suggest that genes were acquired repeatedly by divergent viruses or viral strains of the Nimaviridae.

**Conclusions:**

Our results shed new light on the evolution of the Nimaviridae and point to a long association of this viral group with decapod crustaceans.

**Electronic supplementary material:**

The online version of this article (doi:10.1186/s12862-015-0380-7) contains supplementary material, which is available to authorized users.

## Background

The White Spot Syndrome Virus (WSSV) is a widespread pathogen of several commercially important penaeid shrimp species which causes mass mortalities in aquaculture with high economic impact [1]. The virus is highly virulent, but can also induce latent and asymptomatic infections, which makes creation of virus-free penaeid stocks challenging [2]. Since its discovery in shrimp farms, the virus was detected in a number of other marine and even freshwater decapods: caridean shrimps, brachyuran crabs, anomurans, lobsters and freshwater crayfish [3–7].

WSSV has one of the largest genomes among the viruses that infect animals, with a single circular DNA molecule of about 300 kbp in size [8, 9]. Several lineages of WSSV were described from different geographic locations, with a considerable variation in certain genomic regions, including repeat number variation and deletions leading to differences in genome sizes [10]. Nevertheless, this variation affects only a small part of the genome, while 99.3 % remain identical [10]*.* Thus, all WSSV strains known to date belong to the same viral species, formally classified in the genus *Whispovirus* and a monotypic family – Nimaviridae [1, 10, 11]. This fact is especially noteworthy in the light of the isolated position of this group among DNA viruses as revealed by phylogenetic reconstructions based on DNA polymerase and protein kinase amino acid sequences [12, 13]. Moreover, among the approximately 180 genes identified in the large WSSV genome only a minor fraction shows similarities to already described proteins in other viruses or cellular organisms [1, 8, 9]. About one third of the gene products have been functionally characterized in direct experiments or homology-based bioinformatic approaches: proteins involved in cellular functions (e.g. DNA polymerase, helicase, protein kinases etc.), virion proteins, gene products with proposed roles in latency and early phase of infection and others [1, 8, 9, 14].

The description of new representatives of this phylogenetically distinct viral group is important to allow for evolutionary studies of the Nimaviridae and could advance the understanding of host-pathogen interactions in this system.

Here we report the discovery of a WSSV-like viral genome in a genomic library from the brachyuran crab *Metopaulias depressus* Rathbun, 1896 that is endemic to Jamaica and lives and breeds in water axils of bromeliads [15, 16]. Our results suggest that these viral sequences, together with fragments detected in systematic screenings of previously published decapod genomic libraries [17, 18], stem from several related viral species divergent from WSSV and provide valuable information on the evolutionary history of Nimaviridae.

## Methods

### Genome assembly

The detailed description of the procedures and results of 454-Roche shallow sequencing of a genomic library of *Metopaulias depressus* has been published elsewhere [19]. Briefly, DNA was extracted from muscle tissue using Qiagen DNA Blood and Tissue Kit and 5 μg of it was sent to Macrogen Inc. (Seoul, South Korea) for library preparation and sequencing. The library was pooled together with other libraries and sequenced on a 454 GS-FLX sequencer (Roche). For the crab library 186,890 reads were yielded with an average length of 265.5 base pairs (bp). Our initial estimate of the amount of viral DNA in the data was roughly 10 % and this was the only library among the analyzed crustacean species in which WSSV-like sequences were detected [19]. To identify as many reads belonging to the viral genome as possible the following approach was utilized. The whole dataset was assembled using MIRA v. 3.2.1.5 [20] using relaxed parameters (--job=denovo,est,draft,454, -CO:asir=yes -SK:mnr=no 454_SETTINGS -ED:ace=yes -AL:mrs=75:mo=15 -SK:pr=75) and the resulting contigs were used in BLASTx v. 2.2.29+ [21] (E-value threshold: 0.01) and BLAT v. 35x1 [22] (-t=dnax -q=dnax) searches against the genome of the WSSV isolate from Thailand (AF440570). All contigs matching to the WSSV genome were isolated and associated reads were reassembled with more stringent parameters (MIRA: --job=denovo,est,accurate,454), yielding 918 contigs with 15 of them exceeding 5 kbp. To obtain more accurate information on the consensus sequences of the scaffolded contigs, all reads were mapped back to the chosen MIRA contigs using a local alignment algorithm with Bowtie2 v. 2.0.4 (settings: “--local --sensitive”) [23]. The resulting output files were further processed by custom scripts: all soft-clipping regions left by Bowtie2 were trimmed and all alignments with >80 % of the length covered by short tandem repeats (STRs) were discarded; STRs were detected using Phobos v. 3.3.12 [24] with the default parameters adjusted to minimal STR perfection of 80 % and a maximum unit length of six. Twelve contigs at least 8 kbp long were chosen for scaffolding, while another contig (10 kbp) showed an anomalous coverage profile (presumably mis-assembled) and was not used during scaffold construction. Scaffolding was performed manually based on sequence similarity and gene annotations with subsequent verification of the existence of the overlaps with PCR and Sanger sequencing in questionable cases. The same verification approach was utilized for low coverage regions (<10x per base coverage). Respective primers (Additional file 1: Table S1) were designed with Primer3 v. 2.2.3 [25]. One of the smaller contigs (c152) was later included to extend scaffold I based on the presence of an overlap spanning fragments of two ORFs (homologs of wsv119 and wsv035).

### Reference sequences

The three available WSSV isolates and their genomes are referred to as WSSV-CH (an isolate from *Penaeus japonicus*, China, accession number AF332093; 305,108 bp [9]), WSSV-TH (originating from *Penaeus monodon*, Thailand, AF369029; 292,967 bp [8]) and WSSV-TW (from *Penaeus monodon*, Taiwan; AF440570, 307,287 bp [13]). As the Chinese isolate was previously chosen as the type strain [1], in the current paper we utilize the gene notation system accepted for WSSV-CH with consecutive numbers prefixed by “wsv”.

### Annotation

Potential protein-coding ORFs and their fragmented copies were searched for with BLAT and BLAST. BLAT searches for protein sequences coded by all 184 ORFs recognized in the WSSV-TH genome [8] against the assembled contigs were performed with default settings. BLASTx queries against the GenBank nr1 database (accessed: June 2014) were performed with default parameters and a relaxed E-value threshold of 0.1. In addition, regions between the obtained annotations and unscaffolded contigs were inspected with local BLASTx searches against protein sequences of all potential 532 ORFs from the genome of WSSV-CH with an E-value threshold of 0.01. Finer alignments and detection of frameshifts were carried out by inspection of predictable ORFs with eukaryotic start and stop codons and by performing pairwise translation alignments with ORFs from the WSSV genomes in MACSE v. 0.9b1 [26].

Some occurrences of supposed frameshifts and ambiguous gene boundaries were further verified with Sanger sequencing as described above (see Additional file 1: Table S1).

For subsequent analyses, protein sequences for the annotated loci were predicted based on the standard eukaryotic genetic code. For this purpose, frameshifts were corrected manually. Resulting amino acid sequences were aligned to respective sequences from the WSSV-CH or WSSV-TH genomes in MAFFT v. 7.017 (G-INS-i mode) [27].

### Protein domain prediction

Conserved protein domains were identified based on the amino acid alignments and InterProScan searches [28]*.*

### Interspersed repeats

RECON v. 1.05 [29] was used to predict interspersed repeats. Results from a BLASTn search using the discontiguous-megablast algorithm and an E-value threshold of 10^−5^ were used as the input for RECON. To facilitate comparisons, the same analysis was performed for the genome of WSSV-TH.

### Reanalysis of WSSV-like sequences from shrimp genomic libraries

Repetitive sequences containing WSSV-like fragments from a fosmid [18] and a BAC [17] library prepared from two penaeid shrimp species (*Penaeus monodon* and *P. japonicus*) were downloaded from the *Penaeus* Genome Database (http://sysbio.iis.sinica.edu.tw/page/) and GenBank (accession number AP010878), respectively. The sequences from the fosmid library were represented by 103 repetitive element families with a maximum length of 24 kbp, while the BAC library comprised three fragments of 77, 32 and 9 kbp in length. To locate the WSSV-like genes, BLASTx searches were performed against all ORFs from the WSSV-CH genome with an E-value threshold of 0.01. dUTPase and IAP genes annotated in the two publications were included in the downstream analyses irrespective of their direct association with WSSV-like sequences. Gene boundaries were determined based on ORF locations and homology to WSSV and *Metopaulias* viral genes.

### WSSV-like sequences from EST libraries

To search for WSSV-like sequences in the NCBI non-human and non-mouse EST database (accessed: February 2015) a tBLASTn homology search was used. On that account, protein sequences of all 184 accepted genes from WSSV-TH plus one ORF from the WSSV-CH assembly with homologs in *Metopaulias* (wsv419, see Results) were used as queries. The BLAST results were filtered with an E-value threshold of 10^−5^. In this way, detected EST-sequences were subsequently used as BLASTn and BLASTx queries against the NCBI nt and nr databases (accessed: February 2015), respectively. Sequences that had WSSV genes as their best BLASTx hits with an E-value cutoff of 10^−5^ and at the same time no BLASTn hits, i.e. showing similarity to WSSV on the protein level only, were regarded WSSV-like.

### Protein diversity and similarity

All homologous genes from the decapod genomic libraries were traced and aligned with their orthologs in the WSSV genome using MAFFT (G-INS-i mode). To calculate comparable estimates of protein identity all positions containing at least one gap, unknown amino acid, stop codon or mis-translated regions caused by frameshifts were excluded. In cases of incompatibly truncated protein sequences several alignments were produced for the same gene. Similarity of viral proteins from the decapod libraries and the WSSV-genome was visualized as a hive-plot in HivePanelExplorer (https://github.com/sperez8/HivePanelExplorer) with the axes corresponding to the different genomes, orthologs represented as nodes and edges connecting them reflecting homology and the degree of similarity. The set of genes chosen for WSSV included 106 genes with reported function [1] and/or homology in the decapod libraries. All protein kinases from the decapod libraries were treated as homologs of wsv423 (PK1) (see Results). The ORFs wsv064 and wsv065 were treated as one gene following the evidence from WSSV-TH, WSSV-TW and the *Metopaulias* scaffolds.

### Synteny analysi*s*

Synteny analysis was based on the comparison of the positions of the annotated genes in the *Metopaulias* scaffolds of viral origin and their orthologs in the WSSV genome, and visualized in the program MizBee [30]. To avoid interference of potential mis-assembly, scaffold III was conservatively broken at the low-coverage region (see “Genome assembly” in Results) and at two locations with insertions of large interspersed repeat units (see “Interspersed repeats” in Results).

To facilitate cross-comparison to the shrimp genomic libraries, which are represented by short fragments encompassing only a few genes per fragment, we utilized a breakpoint-oriented approach as defined by Sankoff and Blanchette [31] to summarize topological differences. In short, for all genomes involved in the comparison only shared genes were retained and the remaining pairs of neighboring genes were traced. If presence or absence of a certain gene pair could not be confirmed in at least one fragmented genome, the pair was discarded. A script implementing this breakpoint-oriented approach (breakpoints.pl) is available under https://github.com/har-wradim/miscngs. In the *P. monodon* fosmid library several homologs of the same WSSV-genes were found (see Results) of which only the homologs on the longest respective genome fragments were considered for the breakpoint analysis.

### Phylogenetic analysis

For phylogenetic reconstructions we chose all non-structural genes found among the sequences of viral origin in *Metopaulias* except for those present in WSSV only. Thus, phylogenetic affinities of WSSV and the WSSV-like sequences from the decapod libraries were analyzed based on alignments of the genes coding for DNA polymerase, dUTPase, non-specific endonuclease, helicase, protein kinases (PKs), ribonucleotide reductase subunits (RR1 and RR2), TATA-box binding protein (TBP) and inhibitor of apoptosis (IAP). To identify potential closely related proteins from GenBank (accessed: July 2013), BLASTp searches were performed using WSSV and WSSV-like protein sequences from *M. depressus* as queries with an E-value threshold of 0.01. The best hits (up to 500) were further filtered based on the taxonomic proximity and pairwise identity values using MAFFT alignments: single sequences were retained for groups of proteins of high similarity from closely related organisms. After filtering, new MAFFT alignments were performed using either the G-INS-i mode for proteins with global homology (dUTPase, BIR and RING domains of IAP, RR1, RR2) or the E-INS-i mode for multi-domain proteins (all other cases) with all other parameters being set to default values. Blocks of ambiguous homology (mainly gaps between conserved domains) were excised manually. Maximum-likelihood phylogenies were reconstructed in RAxML v. 7.2.7 [32] with “PROTGAMMALG” as the selected substitution model. Bootstrap support values were estimated based on 500 permutations. Since for different genes incompatible lists of organisms were collected with BLAST and since the resulting topologies sharply diverged, a concatenated dataset was not analyzed.

### PCR assay

To assess infection status in *M. depressus* specimens two fragments were chosen for a PCR assay: a fragment from the homolog of the WSSV main nucleocapsid protein VP664 (wsv360) (primer pair VP664:2 in Additional file 1*:* Table S1) and a fragment of the DNA polymerase gene (wsv514) (primer pair DNApol:4). DNA was extracted from muscle tissue for 22 crabs sampled from different locations across Jamaica using the Puregene kit (Gentra Systems) (coordinates of the sampling sites are available on request). DNA integrity was confirmed by PCR of host multicopy genes (data not shown). Negative controls of the PCR mix without template were run in every PCR reaction to control for contamination of the PCR reagents.

## Availability of supporting data

The sequences of the assembled viral scaffolds from *M. depressus* are available in NCBI GenBank (accession numbers KR820240-KR820242). The raw reads are accessible from the NCBI Sequence Read Archive (BioProject PRJNA283742).

## Results

### Viral sequences in the crab genomic library

#### Genome assembly

*De novo* assembly of the *M. depressus* genomic library resulted in 563 contigs (max. contig size 32,245 bp; N50 = 1,931 bp) including 20,563 reads. Of these, 13 contigs were chosen for semi-manual scaffolding. These contigs were composed of 14,972 reads (8.01 % of the whole genomic library) and the mean coverage achieved 34.1x per site after read mapping. Three scaffolds with lengths of 100,139, 62,154 and 46,520 bp (208,813 bp in total, GC content 44.1 %) successfully incorporated all 13 contigs of putative viral origin. Nevertheless, considerable variation was observed between different reads in terms of single nucleotide polymorphisms and length variation of STRs. Most of the low-coverage and contig junction regions were confirmed with PCR and Sanger sequencing with the exception of one low-coverage region on the scaffold III (primer pairs C9:1 and C9:2: see Additional file 1: Table S1). It was also not possible to fill gaps between the scaffolds.

#### Reconstructed ORFs

Overall the three scaffolds encompass 67 different ORFs (or their fragments) with homology to WSSV (Table 1, see also Additional file 1: Table S2). Unlike all other ORFs, the homolog of wsv294 was initially identified based on positional evidence only: no BLAST hits with an E-value threshold of 0.01 were obtained for its amino acid sequence, but its conserved position between homologs of wsv295 and wsv293a and moderate similarity to wsv294, identified via pairwise alignment justify the homologization. In addition, one ORF showed high similarity to inhibitors of apoptosis proteins (IAPs) from a number of animal species, but not to known WSSV proteins (see below). Alignments of the annotated ORFs with their WSSV homologs suggest that most of them are intact and of full length (see Table 1). The cases of internal frameshifts tested with Sanger sequencing (21 positions) were exclusively associated with either common 454-sequencing artifacts (i.e. homopolymer effects: [33])) or polymorphic STR. In seven cases the predicted protein sequences were considerably shorter than their counterparts in the WSSV genome, of which five were confirmed independently with conventional Sanger sequencing. One of the confirmed truncated ORFs (homolog of wsv293a), if functional, seems to utilize a non-canonical starting codon ACG.

**Table 1:** Orthologs of WSSV genes identified on the three viral scaffolds of *Metopaulias depressus*

Scaffold	Position, bp	Strand	Length, bp	Predicted protein length, aa	ORF number in WSSV ^*b*^			Protein in WSSV				Truncated ends	Frameshifts ^*g*^	MAFFT alignment	
					CN	TH	TW	Family ^*c*^	Protein length	Function ^*d*^				Pairwise identity, %	Alignment length, aa
I	2,517-3,356	-	840	279	299	147	355		309					21.1	341
I	3,524-5,980	+	2,457	818	303	149	359		891	TATA box binding protein (TBP)				48.3	894
I	8,084-12,361	-	4,278	1,425	151	89	207		1,436	Latency-related				26.8	1,538
I	13,933-17,373	+	3,441	1,145	11	36	67		1,301	VP53A, VP150	E	3’		41.2	1,326
I	19,141-21,198	+	2,058	685	220	112	275		674	VP674, VP76, class I cytokine receptor	C			37.6	703
I	22,400-24,835	-	2,436	811	172	92	228		848	Ribonucleotide reductase large subunit (RR1)				55.4	850
I	24,958-28,317	+	3,360	1,119	166	91	222	(NA)	1,072					35.0	1,166
I	29,688-35,294	+	5,607	1,868	447	9	507		1,936	Helicase				43.5	1,986
I	36,408-43,088	+	6,681	2,227 ^*a*^	143	84	198		2,314				Poly	22.9	2,509
I	43,108-44,328	+	1,221	406	188	98	243		413	Ribonucleotide reductase small subunit (RR2)				47.3	442
I	45,135-45,998	+	864	288	(112)	(71)	(168)		462	dUTPase				22.8	468
I	46,200-47,384	-	1,185	394	446	7 + 8	506		526					19.4	563
I	47,874-52,274	+	4,401	1,466	465	16	524		1,243	VP136B				19.3	1,546
I	52,821-55,352	-	2,532	843	45	43	102		981					35.1	1,020
I	55,376-58,807	-	3,432	1,143	37	42	94		1,279	VP160B	C			49.2	1,309
I	59,002-61,614	+	2,613	870	119	73	175		1,044					32.8	1,084
I	61,759-62,034	+	276	91	35	41	92		972	VP110	E	5’, fragment ^*e*^		50.0 ^*i*^	973 ^*i*^
II	898-18,972	-	18,075	6,024	360	167	419		6,077	VP664	C			50.6	6,221
II	19,204-31,302	+	12,099	4,031	343	165 + 166	399 + 412		4,180					38.0	4,331
II	31,716-34,289	+	2,574	857	423	2	482		730	Protein kinase 1 (PK1)				35.0	888
II	34,316-34,906	-	591	196	421	1	480		204	VP28	E			25.5	204
II	35,421-38,787	+	3,367	1,120 ^*a*^	139	82	194		1,208				STR	39.8	1,247
II	38,756-39,691	+	936	311	137	81	192		337	VP337				25.4	351
II	40,142-41,488	-	1,347	448 ^*a*^	131	77	186		484				Poly	33.4	495
II	41,511-42,371	+	861	286	133	78	188		271					33.4	302
III	561-848	-	288	95	21	37	77		200	*hr*-binding protein		5’; 3’ ^*e*^		15.7	204
III	927-1,754	-	828	275	23	38	79		286					37.2	289
III	1,820-2,215	-	396	131	25	39	81		231			5’ ^*e*^		16.2	247
III	2,231-6,865	-	4,635	1,543	26	40	86 + 82		1,535	VP507				50.2	1,590
III	8,195-11,185	-	2,991	996	226	114	281		936					27.2	1,074
III	11,727-14,753	-	3,027	1,008	192	100	247		1,019					44.8	1,051
III	14,831-15,739	+	909	302	191	99	246		311	Endonuclease				44.9	325
III	17,211-18,929	-	1,719	572	427	3	486		623	Latency-related				34.2	658
III	19,167-22,964	+	3,798	1,265	433	4	492 + 496		1,261					51.6	1,323
III	23,005-24,687	+	1,683	560	440	5	500		607					46.2	623
III	24,642-27,239	+	2,598	865	442	6	502		800	VP800, VP95	T			30.4	895
III	30,327-33,310	-	2,984	994 ^*a*^	64 + 63	53	121 + 120		843				Poly	16.4	1,099
III	38,213-40,172	-	1,960	653	277	135	332		791					31.6	807
III	40,595-44,461	-	3,867	1,289	271	134	326		1,219	VP136A	C		Poly	36.1	1,348
III	44,426-44,971	+	546	181	270	133	325		170					34.2	190
III	44,938-46,377	+	1440	479	269	132	324		489	VP53c				53.9	505
III	46,469-47,209	+	741	246	267	131	321		281					28.1	281
III	47,325-49,637	+	2,313	770	260	130	315		922	VP387				25.3	965
III	50,920-52,581	-	1,662	553	1	30	52		1,684	VP1684, collagen	E			23.5 ^*i*^	613 ^*i*^
III	52,725-55,115	-	2,391	796	332	160	388		786	VP75				38.2	824
III	55,224-57,680	+	2,457	818	327	159	383		856	VP90	E			40.1	872
III	58,441-59,865	+	1,425	473	147	85	202		459					19.8	515
III	62,523-63,143	-	621	206	419	(NA)	478		193					31.9	210
III	62,963-64,516	-	1,554	517	415	183	474		544	VP544, VP60 §	C			43.0	547
III	64,652-65,005	+	354	117	414	182	473		121	VP19	E			39.3	122
III	65,332-65,604	+	273	90	324	157	380		82					31.3	98
III	65,906-66,142	-	237	78	322	156	378		227			5’, 3’; fragment		16.1 ^*i*^	227 ^*i*^
III	66,363-66,752	-	390	129	321	155	377		117	VP13B, VP16	E			39.5	129
III	66,908-69,883	+	2,976	991	313	154	369		1,179					29.9	1,224
III	69,974-70,567	+	594	197	311	153	367		204	VP26	T			28.2	206
III	70,645-71,418	-	774	257	310	152	366		272					33.0	277
III	71,415-72,818	-	1,404	467	308	151	364		466	VP466, VP51	C			44.4	480
III	72,809-73,111	-	303	100	306	150	362		419	VP39A	T	5’ ^*e*^		10.8	435
III	73,422-75,302	+	1,881	626	282	140	337		634					54.1	658
III	75,443-77,426	+	1,984	660 ^*a*^	161	90	217		759				Poly	29.8	786
III	77,503-84,399	+	6,897	2,298	514	27	39		2,351	DNA polymerase				55.0	2,409
III	84,915-86,372	+	1,458	485	no homology					Inhibitor of apoptosis protein					
III	86,662-87,348	-	687	228	295	146	351		209					23.6	267
III	87,428-87,922	-	495	164	294	145	350		206					17.6	216
III	88,010-88,258	+	249	82	293a	144	349		97	VP14		5’ ^*ef*^		55.3	97
III	88,215-92,825	+	4,611	1,536	289	143	344		1,564	VP160A, VP190	C			38.1	1,627
III	93,236-96,400	-	3,165	1,054	285	142	340		1,100					36.9	1,116
III	96,852-98,046	-	1,195	398 ^*a*^	78	59	135		398	*hr*-binding protein			Poly	20.9	460

^*a*^based on protein sequences after “correction” of the frameshifts

^*b*^Accession numbers for the three reference genomes see in text. ORF identification numbers are taken either from original publications or from GenBank annotations. Cases of frameshifts in the WSSV genomes are marked with “+” connecting two parts of the original ORF. “(NA)” marks an ORF present in WSSV-TH genome, but not annotated

^*c*^families of paralogs according to [8]. “(NA)” marks one family not reported in that study

^*d*^based largely on the summary presented by [1]. Structural proteins are prefixed with “VP”, their location (when known) is marked with letters: C – nucleocapsid; T – tegument; E – envelope

^*e*^cases of truncations confirmed with Sanger-sequencing

^*f*^additionally: predicted start codon is non-canonical (ACG)

^*g*^causes of frameshifts (when present, presumably sequencing artefacts): Poly – homopolymer regions; STR – short tandem repeats

^*h*^all alignments were performed with the G-INS-i mode. If not noted otherwise, identities are reported for the whole alignments

^*i*^based on truncated alignments

Thirty-six (53.7 %) of the reconstructed genes are homologous to previously functionally characterized genes of WSSV (and other organisms in the case of IAP. Out of the 21 non-structural genes reported for WSSV [1, 2, 8, 34, 35] twelve were found on the reconstructed *Metopaulias* scaffolds. These include most of the proteins involved in replication and nucleotide metabolism: the DNA polymerase, helicase, non-specific nuclease, dUTPase and both subunits of the ribonucleotide reductase, while no homologs of the chimeric thymidine/thymidylate kinase and the thymidylate synthase were found. Additionally, homologs to both homologous-region-binding proteins were identified: one of them (wsv021: [36]) is represented by a considerably truncated ORF in *M. depressus*, while the second one (wsv078: [34]) is presumably of full length (see Table 1).

Out of the 17 major virion proteins currently recognized in WSSV [1] we found homologs of genes coding for nine of them: VP664, VP160B, VP51C (nucleocapsid); VP95, VP39A, VP26 (tegument); and VP53A, VP28, VP19 (envelope). An intact ORF with a predicted amino acid sequence similar to the collagen protein from WSSV (wsv001, VP1684) was also identified, but contained much less of the tandem collagen tripeptide (Gly-Xaa-Yaa) repeats (388 vs. 26 units).

Among the 550 unscaffolded contigs, 344 had BLASTx hits when searched for the protein sequences from WSSV. Besides the homologs of WSSV genes already discovered on the scaffolds, four homologs to additional genes were identified after filtering (Table 2). None of these WSSV proteins were previously functionally characterized.

**Table 2:** Fragments homologous to WSSV ORFs identified only on non-scaffolded viral contigs from Metopaulias

ORF number in WSSV			Number of contigs	Longest match ^*a*^		
CH	TH	TW		E-value	Match length, bp	Identity, %
4	32	60	1	8.3E-04	153	31.4
91	65	148 + 149	1	1.9E-03	84	42.9
178	94	234	1	6.9E-03	222	27.6
79	60	136	6	3.5E-27	525	31.1

^*a*^ The matches are based on local BLASTx searches against all ORFs from WSSV-CH, with subsequent removal of hits to minor “internal” ORFs and those having a higher similarity to other genes from WSSV

Analysis of the BLASTx hits for all contigs taken individually revealed that the vast majority of WSSV homologs were represented by more than one fragment (median: 3; 5-95 % quantiles: 1–19). Although the relative contribution of contig overlaps, mis-assembly and true multiple insertions could not be clearly disentangled, there were seven outliers with more than 19 fragments each, representing different parts or sequence variants of the respective ORFs (Table 3).

**Table 3:** Homologs of WSSV genes represented by more than 20 fragments on scaffolds and/or unscaffolded contigs

ORF number in WSSV			Number of fragments
CH	TH	TW	
1	30	52	108
360	167	419	67
191	99	246	66
415	183	474	65
327	159	383	62
343	165 + 166	399 + 412	48
514	27	39	30

#### Interspersed repeats

**Fig. 1:**
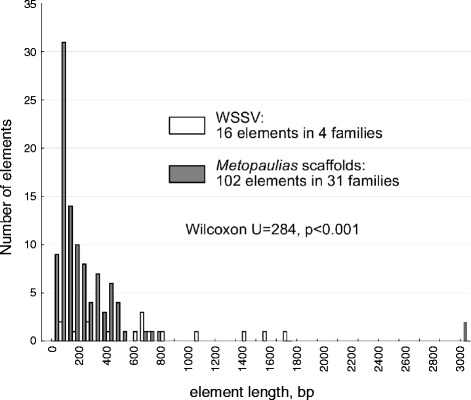
Frequency distributions of lengths of individual units in families of interspersed repeats as identified by RECON in the genome of WSSV and scaffolded viral sequences from *Metopaulias depressus*

A screen for interspersed repeats was performed both for the WSSV genome and the *M. depressus* viral scaffolds (Fig. 1). Most of the repeated regions in WSSV correspond to the previously identified “homologous regions” *(hrs)* (see [35]). The *M. depressus* scaffolds possess significantly shorter, but one order of magnitude more abundant repetitive elements. Nevertheless, no repeats similar to the *hrs* of WSSV in sequence or structure could be identified.

The longest identified interspersed repeat (see Fig. 1) corresponds to a region on scaffold III intersecting intact homologs of wsv327 and wsv147 interrupted by a fragment homologous to wsv191, and a similar region with broken copies of all three ORFs on scaffold II. Among WSSV homologs represented multiple times the homologs to wsv327 and wsv191 were detected at least thrice: wsv327 with five fragments of varying lengths, one of them being the intact ORF neighboring a homolog of wsv332 (the adjacent ORF in the WSSV genome); and wsv191 (the endonuclease) represented by three different copies, one of them being the intact ORF neighboring a homolog of wsv192.

#### Transposon insertions

**Fig. 2:**
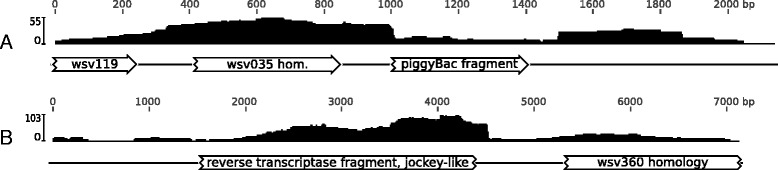
Examples of contigs containing transposons in *Metopaulias depressus* with respective per-base coverage graphs shown above. (**a**) Contig c152 (partly incorporated in scaffold I) with a fragment homologous to wsv035 and a 3’-fragment of piggyBac transposase. (**b**) Contig c14 (not scaffolded) with a fragment similar to reverse transcriptase of jokey-like transposons and a fragment with homology to wsv360

Fragments of transposon proteins were identified in 36 contigs, with the majority of them belonging to reverse transcriptase from jockey-like retrotransposons (data not shown). In 21 cases these contigs contained also fragments similar to WSSV ORFs (Fig. 2). One of the contigs containing a transposon-like sequence (3’-terminal fragment of the piggyBac ORF) was incorporated at the end of scaffold I (Fig. 2b). Abrupt coverage drop-out indicates that the transposon-like sequence is not present in all of the copies of the respective region, which was confirmed by a manual inspection of alternative assembly paths. Based on this information the region beyond the coverage decline point was discarded during preparation of the final scaffold sequence.

#### Prevalence of WSSV-like sequences in Metopaulias individuals

Our PCR assay was designed on the basis of the homologs of wsv360 (VP664) and wsv514 (the DNA polymerase) represented on the scaffolds. This choice was motivated by the functional significance of these genes in WSSV and apparent lack of variation in the respective fragments in the reference specimen (but see below). The PCR test was positive for all 22 DNA samples from *Metopaulias* individuals from across Jamaica (Additional file 2*:* Figure S1). For twelve individuals the DNA polymerase fragment was Sanger-sequenced in both directions to produce 715-bp sequences. No nucleotide position was variable among individuals, but at least one position appeared to be polymorphic in each one of the sequences: a synonymous A/G variation at position 111, which was also confirmed for the reference specimen (Additional file 2*:* Figure S2).

### WSSV-like sequences in the shrimp genomic libraries

Although the inventories of the WSSV-like genes were provided in the original publications [17, 18], we decided to perform a new analysis similar to the one performed for the *M. depressus* library, primarily to clarify gene boundaries and extract protein sequences. For the *P. monodon* repetitive families our BLASTx search yielded 76 hits (of which 48 were reported in the original study) to 48 different WSSV genes (see Additional file 1*:* Table S3). Seven hits to seven WSSV genes were obtained for the *P. japonicus* BAC library, all of which were originally reported and numbered (genes 11, 13–18) (see Additional file 1*:* Table S4).

Additionally, in both libraries single dUTPase ORFs and several IAP genes were confirmed in locations indicated in the original publications. All genes were represented by single continuous ORFs with the exception of one IAP gene from the *P. japonicus* BAC library, which we interpret as consisting of at least four exons. In contrast to Koyama and co-authors [17], we consider it separately from the IAP gene next to it and designate them as genes 06A and 06B respectively (see below).

### WSSV-like sequences in EST libraries

**Fig. 3:**
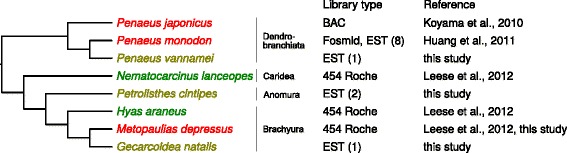
Decapod species with fossilized WSSV-like viruses (marked red), with WSSV-like sequences in ESTs libraries (yellow) or with evidence of absence of WSSV-like sequences in genomes (green). The cladogram to the left is a simplification of the phylogenetic relationships between the species considered based on (Tsang et al. 2008; Ma et al. 2011; Tsang et al. 2014), all other decapod groups are omitted. The numbers of WSSV-like ESTs discovered in our BLAST analysis are provided in parentheses

A conservative search for WSSV-like sequences in all non-human and non-mouse EST libraries available in the NCBI EST database revealed 12 positive hits with similarity to WSSV on the protein level only. These hits were distributed among four species, all of which belong to decapod crustaceans (see Fig. 3).

### Cross-comparison of WSSV and WSSV-like genomes

#### Diversity and similarity of proteins with homology in WSSV

**Fig. 4:**
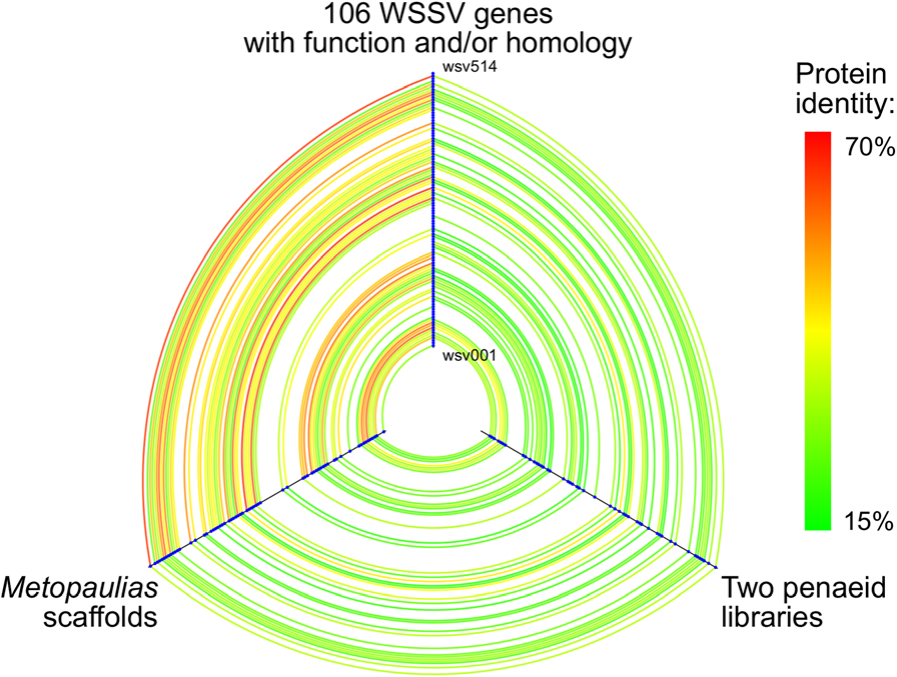
Similarities between WSSV-like genes in the crab and shrimp genomic libraries visualized as a hive-plot. The three axes correspond to 1) genes from WSSV with a reported function (Leu et al., 2009) and/or homology to sequences from the decapod libraries; 2) homologs of WSSV-genes from the *Metopaulias* library and 3) homologs of WSSV-genes identified in the repetitive sequences from *Penaeus monodon* (Huang et al., 2011) and *P. japonicus* (Koyama et al., 2010). Genes on the axes are arranged in the order on the WSSV-CH genome. Lines connecting homologs are colored according to amino acid similarities (see Additional file 3). When multiple alignments were available for the same gene only one was chosen. In cases of multiple paralogs in the penaeid shrimp libraries only one representative was taken with the preference given to proteins from *P. japonicus*

Out of the 48 unique WSSV homologs in the *P. monodon* library, 38 (79 %) were found in the *M. depressus* scaffolds (Fig. 4, Additional file 3). Under the hypothesis of a common ancestor with a genome composition identical to that of WSSV, the probability of obtaining at least this number of matches by chance from the set of 106 WSSV genes with known function or homology equals to 0.0017. Furthermore, if the gene set is expanded to the 180 genes currently hypothesized to be encoded by the WSSV genome [1, 8] the probability decreases to 3.64 × 10^−12^. Of the seven genes with WSSV homology from the *P. japonicus* BAC clone all are identifiable in the other shrimp library and only one (wsv306) is missing from *M. depressus* (see Additional file 3). BLASTx searches revealed no genes shared by the shrimp repetitive sequences and crab scaffolds but absent from the WSSV genome, with the noticeable exception of IAPs (see below).

The proteins from the shrimp libraries appear significantly less similar to their homologs in the two other gene sets in comparison to identity values between WSSV and *M. depressus* with an average difference in *p*-distances of 0.2 (Fig. 4, Table 4). Nonetheless all three sets of pairwise distances are strongly correlated with each other (see Table 4).

**Table 4:** Comparisons of *p*-distances between 38 proteins shared by the WSSV genome (W), *M. depressus* scaffolds (M) and penaeid libraries (P): summary statistics, correlation coefficients and results of Wilcoxon signed rank tests. Asterisks indicate significance levels after Holm’s correction: * – p < 0.05, **** – p < 0.0001

			Pearson’s *r* \ Median difference		
	Mean	SD	PM	WM	WP
PM	0.714	0.0663		0.198****	−0.011*
WM	0.526	0.0899	0.805****		−0.201****
WP	0.720	0.0698	0.898****	0.726****	

Many of the WSSV-homologs are found several times on different fragments in the *P. monodon* library. Due to the fact that many of them are represented by incomplete gene sequences, the exact number of genes with several WSSV paralogs could not be estimated. However, 22 pairs of overlapping fragments representing 19 unique genes could be determined (see Additional file 3) with the mean (±SD) *p*-distance of 0.687 ± 0.0728. No unambiguous cases of more than two overlapping paralogs were found. Twelve fragments from *P. monodon* could be compared to the seven WSSV homologs from the *P. japonicus* library with the resulting mean *p*-distance of 0.593 ± 0.2340.

#### Synteny between WSSV and M. depressus scaffolds

**Fig. 5:**
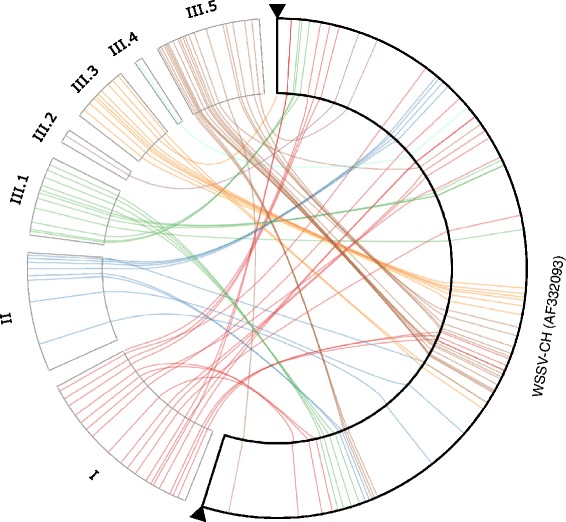
Synteny conservation between the WSSV genome (represented by WSSV-CH) and *Metopaulias depressus* viral scaffolds. The scaffolds are labeled with roman letters, with scaffold III being broken at two points of ambiguous joins (see Material and Methods for details). Lines are colored according to respective *Metopaulias* scaffolds

The gene order on the *Metopaulias* viral scaffolds was compared to the WSSV genome annotations (Fig. 5). Overall, 14 syntenic blocks covering 43 genes were identified. Half of the blocks include just single pairs of neighboring genes, while the largest block spans eight genes (wsv306-wsv324). No cases of inversions were identified in individual blocks: neighboring genes show consistently the same orientations in both genomes.

#### Analysis of breakpoints

In order to enable comparison of gene orders with the data from the shrimp libraries, we utilized a breakpoint-oriented approach. For the set involving WSSV, *M. depressus* scaffolds and *P. monodon* repeat families, 50 pairs of neighboring genes could be inspected for their presence/absence. Resulting breakpoint distances were nearly identical for all three pairs of viral genomes: WSSV genome vs. crab scaffolds – 28 breakpoints, WSSV genome vs. shrimp library – 28 breakpoints, crab scaffolds vs. shrimp library – 30 breakpoints (no statistical test performed). In total only seven neighboring gene pairs were shared by all three gene sets (the “-” sign denotes genes on the opposite strand): wsv023/wsv021, -wsv327/wsv332, wsv306/wsv308, wsv293a/wsv289, wsv139/wsv137, -wsv427/wsv433 and wsv433/wsv440. Due to the low number of genes n the fragment containing WSSV-like sequences from *P. japonicus*, its addition decreased the number of gene pairs amenable to the analysis to ten with only a single pair of neighbors shared by all four gene sets: -wsv327/wsv332, which was also the sole pair shared by *P. japonicus* and *P. monodon*.

### Phylogenetic analyses

#### DNA polymerase

**Fig. 6:**
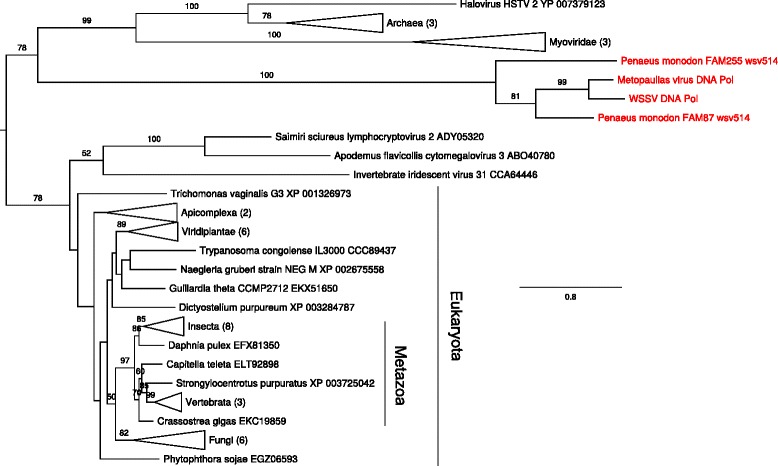
Maximum likelihood (ML) phylogenetic tree for DNA polymerase amino acid sequences (alignment length 833 residues). The tree is formally rooted on the split between Eukaryota and Archaea. Bootstrap support values ≥ 50 % are indicated above the branches. The proteins from WSSV and from the three decapod genomic libraries are in red

Single genes coding for DNA polymerases were found in WSSV and in the *M. depressus* library, while the *P. monodon* library contained two distinct polymerases. Phylogenetic analysis shows three well-supported major clades corresponding to polymerases from 1) eukaryotes and three viral groups; 2) archaeans, Myoviridae and a halovirus; 3) and the WSSV and WSSV-like polymerases (Fig. 6). Within this last clade the two polymerases from *P. monodon* do not cluster together and occupy basal positions in relation to the two other polymerases (see *also* Additional file 2: Figure S3).

#### Helicase

The WSSV genome and the *M. depressus* scaffolds contained single-copy helicase genes each, while two distinct helicase sequences are identifiable in the *P. monodon* library. The helicases from WSSV and the decapod libraries cluster in a well-supported clade presumably at the base of eukaryotic helicases while bacterial helicases form a distinct clade (predominantly Bacteroidetes) (see Additional file 2*:* Figure S4). Among the four helicases of viral origin the sequences from *P. monodon* occupy basal positions and do not form a clade with each other.

#### Ribonucleotide reductase subunits

**Fig. 7:**
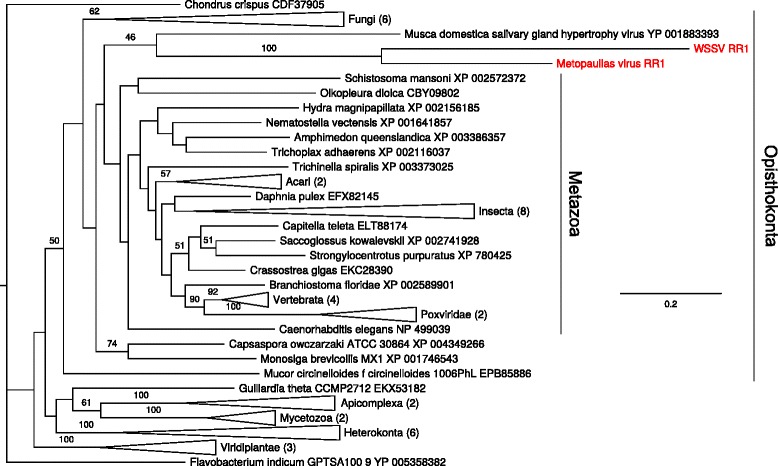
ML phylogenetic tree for ribonucleotide reductase large subunit (RR1) amino acid sequences (alignment length 769 residues). The tree is rooted by the sequence from *Flavobacterium indicum* (Bacteria)

The phylogenetic tree reconstructed for the RR1 gene is largely poorly resolved (Fig. 7, see also Additional file 2*:* Figure S5). The RR1 proteins from WSSV and *M. depressus* cluster together within a large clade covering opisthokonts. Results for the RR2 alignment show a very similar pattern with similarly low resolution (see Additional file 2*:* Figure S6).

#### TATA-box binding protein

**Fig. 8:**
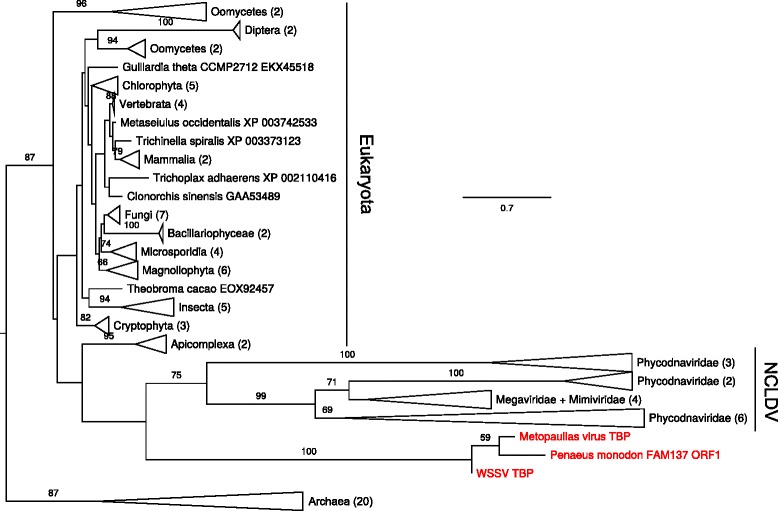
ML phylogenetic tree for TATA-box binding protein (TBP) amino acid sequences (alignment length 173 residues). The tree is formally rooted by the sequences from Archaea

Phylogeny reconstructed for the TBP sequences shows the proteins from WSSV and *M. depressus* to branch off close to viruses from the NCLDV group with only a weak support (Fig. 8, see also Additional file 2*:* Figure S7).

#### Endonuclease

**Fig. 9:**
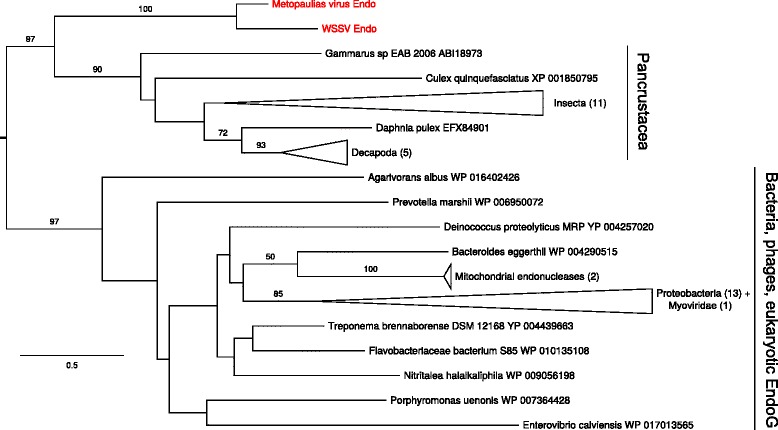
ML phylogenetic tree for endonuclease amino acid sequences (alignment length 144 residues). The root corresponds to the branching of of endonucleases bearing eukaryotic signal-peptide: see Wynant et al. (2014)

The viral endonucleases from WSSV and *M. depressus* both possess a eukaryotic signal peptide and cluster together at the base of double-stranded ribonucleases bearing similar signal peptides identified in crustaceans and insects (Fig. 9, see also Additional file 2*:* Figure S8).

#### Protein kinase

Among the two WSSV PK enzymes the sole protein kinase identified in *M. depressus* is presumably an ortholog of WSSV PK1, given the higher similarity and shared location of the ORF next to the homolog of wsv421. The two kinases from the *P. monodon* library are roughly equally distant from WSSV PK1 and PK2 and from each other (see Additional file 3) and no positional information is available for them. The alignment yielded a tree with poor resolution around the five protein kinases (see Additional file 2*:* Figure S9). Nevertheless, they form a distinct clade in the PK tree with high bootstrap support. The two *P. monodon* proteins group with WSSV PK2, but do not form a cluster with each other, while WSSV PK1 clusters with the sole PK sequence from *M. depressus* with weak support. Among the PK-sequences sampled with BLASTp, serine-threonine kinases were the only class of PKs with explicitly specified activity.

#### Inhibitor of apoptosis proteins

The tree for the BIR-domains of the IAPs associated with WSSV-like sequences in the decapod libraries and IAPs from different animal taxa is poorly resolved due to the very low number of positions (72 amino acids) (Additional file 2*:* Figure S10). Still, three or four large clades involving IAPs from the three decapod libraries and four previously identified IAPs from penaeids and a caridean shrimp are discernible and roughly correspond respectively to the first, second and third BIR domains of the decapod three-BIR-domain IAPs. Three second BIR domains from the shrimp repetitive sequences form a separate clade close to the clade of the first BIR domains. The IAP from *M. depressus* has only two BIR domains: one of them clusters together with the group of second domains of other IAPs, and the other one does not belong to any of the three decapod clades. Another outlying BIR domain is the third BIR domain of the IAP coded by gene 06A from the *P. japonicus* library. Not all of the IAPs contain RING domains, and the phylogeny reconstructed based on the respective alignment (35 positions only) shows a grouping of the RING domains from two IAPs from the two shrimp libraries together with IAPs independently identified in penaeid and caridean shrimps (see Additional file 2*:* Figure S11). The RING-domain from the *M. depressus* IAP does not belong to this clade.

#### dUTPase

The phylogenetic tree based on the alignment for the dUTPase protein sequences also lacks resolution (see Additional file 2*:* Figure S12). The four dUTPases from WSSV and the three decapod libraries do not form a single monophylum and cluster together with other sequences in different parts of the tree, albeit with low support values. At the same time, the dUTPases from the two shrimp libraries are closely related and form a well-supported grouping.

## Discussion

The White Spot Syndrome Virus (WSSV) is currently considered as the sole representative of an isolated viral group with unclear relationships to other viruses [1, 8]. In this study we describe a viral genome with high similarity to WSSV discovered in a genomic library of the Jamaican bromeliad crab *Metopaulias depressus* [19]. The genome is seemingly nearly complete given the fact that undamaged homologs of most of the WSSV non-structural proteins (including the DNA polymerase) and half of the major virion protein genes were identified. Nevertheless, we postulate that this virus is not independent, but endogenized in the genome of its host, because: 1) despite the high sequencing coverage we were not able to reconstruct a single circular molecule as known for WSSV, and no connection between the three reconstructed scaffolds could be detected with PCR, 2) some fragments of WSSV-like genes are clearly not functional, 3) several transposon-derived sequences are found in association with the WSSV-like sequences, 4) there is considerable intra-individual variation in the specimen chosen for sequencing, and 5) analogous variation is obtained in a test fragment sequenced for several other crab specimens from Jamaica. The unexpectedly high ratio of the viral to host DNA (1:10) and intact state of the genes with WSSV homology located on the scaffolds are mirrored by the two similar cases of WSSV-like sequences discovered in genomic libraries of penaeid shrimps [17, 18], that were reanalyzed for this study.

### Relationships between decapod WSSV-like sequences and WSSV

In all three cases of the WSSV-like viruses in decapod genomes, the similarity to WSSV is restricted to the protein level only. Together with the difference in gene order this clearly indicates that these sequences are not derived from contemporary infections by WSSV. Despite the fact that the genomes of the crab and the two penaeids contain very high amounts of viral sequences of similar origin, respective endogenization events must have taken place independently, since no WSSV-like sequences were found by us in two other decapod libraries: from a brachyuran crab and a caridean shrimp [19] (see Fig. 3). Moreover, while *M. depressus* and *P. japonicus* contain single sets of WSSV-like genes in their genomes, *P. monodon* unambiguously shows signs of multiple genome integration events as already pointed out by Huang and co-authors [18]. In addition, our analysis of the EST database shows that the phenomenon of the presence of WSSV-like sequences in genomes (or infection with WSSV-like viruses) might be relatively common among decapods and urges a systematic investigation.

Phylogenetic trees reconstructed for slowly evolving genes and the fact that the viral sequences integrated in the crab genome are much more similar to WSSV genes than their counterparts from the shrimp genomes, indicate that the divergence of the viruses integrated in penaeid genomes predates the split between WSSV and the virus integrated in *M. depressus*.

The gene order in WSSV and in the sequences of viral origin in decapods are very different, although the shuffling is incomplete and syntenic blocks can still be recognized in the scaffolds reconstructed for *M. depressus*. Inspection of shared neighboring genes for the largest gene sets, WSSV, *M. depressus* scaffolds and *P. monodon* repetitive elements, shows that all three viral genomes are equidistant from each other in terms of gene order. Most of these structural differences were likely present in the WSSV-like viruses before their integration in the decapod genomes, because the rearrangements do not seem to involve any host genes in the case of *M. depressus* and only in isolated cases were they involved in the fragments of viral origin in shrimps [17, 18].

### Ancestral gene composition and new gene acquisitions

Despite the structural dissimilarity and prominent differences in homologous protein sequences, the viral genome detected in *M. depressus* on the one hand and the viral sequences found in the shrimp databases on the other show striking similarity in gene content. While the WSSV genome is predicted to encode about ~180 genes, we were able to identify only 67 of their homologs in the *M. depressus* and 48 in *P. monodon*. Of these, homologs for 38 genes were detected in all three gene sets. This indicates that most of the WSSV genes are either 1) lineage-specific, 2) have high evolutionary rates and cannot be detected with the homology-based methods we utilized, or 3) were not recovered due to the selective loss of genes in the fossilized viral genomes. The fact that the majority of ORFs in the viral genome recovered from the *M. depressus* library remain uncharacterized indicates that the comparatively low number of WSSV homologs detected is not due to the incompleteness of the draft genome. In a single case we were able to recover a WSSV homolog thanks to positional information only, indicating low sequence conservation for this gene. The existence of lineage-specific genes is evidenced e.g. by the dUTPase genes. While a single dUTPase gene is present in WSSV [37] and its homologues were also present in the decapod viruses, at least three independent gene acquisition events can be inferred. A similar situation with purported multiple acquirements of dUTPases is known for Siphoviridae [38].

A complex history also underlies the evolution of the inhibitor of apoptosis proteins. While two proteins involved in apoptosis suppression were previously identified in WSSV [39, 40], none of them showed similarity to the IAP family. At the same time a single IAP ORF was found in the *M. depressus* virus and several IAP-coding genes were discovered in repetitive sequences in shrimps, even if not always in clear direct association with WSSV-like sequences [17, 18]. Most of these IAPs group together with IAPs independently identified in penaeid and caridean shrimps [41]. At the same time, the sole IAP from the *M. depressus* scaffolds may be of mosaic origin: only one of its three domains undoubtedly comes from the same source as other decapod IAPs.

### Fossilized WSSV-like viruses and the decapod hosts

The protein similarities between WSSV orthologs are highly correlated indicating that different genes have their own substitution rates, which remained relatively stable since divergence of the viruses. This, and also the absence or relative rarity of frameshifts and premature stop codons in the WSSV-like genes from the decapods can be explained by either their contemporary functionality in the host genomes or very recent genome integration events. While the second scenario cannot be completely excluded, direct evidence does exist in favor of at least some transcriptional activity of the WSSV-like genes in shrimps [17, 18] (see also Fig. 3). It has been hypothesized that these sequences may play a role in defending the host from WSSV infection [18]. It is noteworthy in this respect, that *M. depressus* is a fully terrestrial crab, which evolved from marine ancestors about 4.5 mya and since then had no persistent contact with other decapods [15]. As WSSV is transmitted horizontally between hosts through water [7], it is likely that the WSSV-like virus was integrated into the crab genome before the switch of the host to terrestrial habitats. Otherwise, the same or a similar virus may still be present in the crab population, but in that case it must utilize a different way of transmission.

The amount of WSSV-like sequences in all three decapod libraries is surprisingly high: about 10 % in *M. depressus* and about 22 % in *P. monodon*, which has clearly very important consequences for the genome organization of the hosts [17, 18]. Although some differences between different copies of the same regions of viral origin are identifiable, relative scarcity of this variation must be explained by the action of mechanisms similar to concerted evolution of rRNA gene clusters (see e.g. [42]).

The very fact of the presence of genomic fragments of WSSV-like viruses in host genomes is rather surprising, since WSSV itself does not integrate in the host genome [43]. Nevertheless, similar cases of fossilization of viruses which normally have no integrated stages were discovered in other animals [44–46].

The presence of large genomic fragments from WSSV-like viruses in decapod genomes is indicative of a long term co-evolution and points at the existence of multiple lineages within this viral group. Other non-fossilized members of the Nimaviridae are thus expected to be discovered. One of the practical consequences of this hypothesis is that the methods currently being developed developed to detect and resist the spread of the white spot syndrome in penaeid shrimp aquaculture [47–50] would require adjustments to target multiple species from the same group.

### Phylogenetic position of the Nimaviridae

A wider sample of (endogenized) viruses related to WSSV could potentially bring new information about affinities of this isolated viral group (for earlier attempts see [12, 13, 51]). Although the results are not yet conclusive, it is clear that no other extant virus family is closely related to this group. The DNA polymerase and TBP phylogenies point to a very basal position of the clade within Eukaryota or close to the split Eukaryota-Archaea. Other genes (both subunits of the ribonucleotide reductase and the endonuclease) are seemingly of metazoan or at least opisthokont origin. As already indicated by Wynant and co-authors [52], the WSSV endonuclease belongs to a specific clade of enzymes found exclusively in Pancrustacea (crustaceans and hexapods); its ortholog discovered in this study shares its structure and basal position within this clade.

## Conclusions

Our study gives the first detailed analysis of fossilized relatives of the White Spot Syndrome Virus, an important pest in penaeid shrimp aquaculture with obscure phylogenetic affinities. Genomic fragments of these viruses are found in several decapod species, but reflect independent endogenization events and are not directly related to the contemporary infection with WSSV. In this respect our study provides an important starting point for comparative studies of WSSV.

## Additional files

Additional file 1: Table S1. List of primers utilized for PCR. **Table S2.** Results of blastp searches for the predicted protein sequences from the viral scaffolds of *Metopaulias depressus* against GenBank. **Table S3.** ORFs and ORF fragments with homology to WSSV, dUTPases or Inhibitor of Apoptosis Proteins (IAPs) in the sequences of repetitive families identified in the *Penaeus monodon* fosmid library (Huang et al., 2011). **Table S4.** ORFs and ORF fragments with homology to WSSV, dUTPases or IAPs found in the sequence of the Mj024A04 BAC-clone from *Penaeus japonicus* (Koyama et al., 2010). Additional file 2: Figure S1. Agarose gel electrophoresis of a diagnostic PCR-product for a subset of *Metopaulias* individuals. The product corresponds to a fragment of the wsv360 homolog (primer pair “VP664:2”). The first and the last lanes are size-standards. The penultimate lane is a negative control. **Figure S2.** Polymorphic position on the sequencing chromatograms for a PCR product from the homolog of DNA polymerase. Shown are 17 bases around the A/G silent polymorphism in the fragment amplified with primers VP664:2. Among the 5 individuals represented the bottommost one is the individual, for which the original 454-library was prepared. **Figure S3.** Full ML phylogenetic tree for DNA polymerase amino acid sequences. **Figure S4.** ML phylogenetic tree for helicase amino acid sequences (alignment length 624 residues). The tree is formally rooted on the split between Bacteria and Eukaryota. **Figure S5.** Full ML phylogenetic tree for ribonucleotide reductase large subunit amino acid sequences. **Figure S6.** ML phylogenetic tree for ribonucleotide reductase small subunit amino acid sequences (alignment length 316 residues). The tree is rooted on the split between Bacteria and Eukaryota. **Figure S7.** Full ML phylogenetic tree for TATA-box binding protein (TBP) amino acid sequences. **Figure S8.** Full ML phylogenetic tree for endonuclease amino acid sequences. **Figure S9.** ML phylogenetic tree for protein kinase amino acid sequences (alignment length 227 residues). The tree is formally rooted by the sequences from the main part of Bacteria. **Figure S10.** Full ML phylogenetic tree for BIR-domains of Inhibitor of Apoptosis Proteins. The tree is formally rooted by a clade of predominantly vertebrate sequences. **Figure S11.** ML phylogenetic tree for RING-domains of Inhibitor of Apoptosis Proteins (alignment length 53 residues). The tree is formally rooted by a clade of predominantly vertebrate sequences. **Figure S12.** ML phylogenetic tree for dUTPase amino acid sequences (alignment length 137 residues). The tree is rooted on the split between Bacteria and Eukaryota. Additional file 3:
**XLS spreadsheet summarizing pairwise similarity values (%) between protein sequences shared by WSSV and WSSV-like sequences from the three decapod libraries.** Each sheet corresponds to a single MAFFT alignment. The lengths of the resulting alignments are indicated in the A1-cells. This file served as the input for Figure 3; in cases of multiple alignments per gene only one of them was used, others being labeled with a “no hive” flag.
