# Supplementary material for: Characterization of fossilized relatives of the White Spot Syndrome Virus in genomes of decapod crustaceans
Source: BMC Evol Biol. 2015 Jul 19;15:142. doi: 10.1186/s12862-015-0380-7 (PMC4506587; doi:10.1186/s12862-015-0380-7)
Supplement: Additional file 1: Table S1. — List of primers utilized for PCR. Table S2. Results of blastp searches for the predicted protein sequences from the viral scaffolds of Metopaulias depressus against GenBank. Table S3. ORFs and ORF fragments with homology to WSSV, dUTPases or Inhibitor of Apoptosis Proteins (IAPs) in the sequences of repetitive families identified in the Penaeus monodon fosmid library (Huang et al., 2011). Table S4. ORFs and ORF fragments with homology to WSSV, dUTPases or IAPs found in the sequence of the Mj024A04 BAC-clone from Penaeus japonicus (Koyama et al., 2010). [file 12862_2015_380_MOESM1_ESM.pdf]

Characterization of fossilized relatives of the White Spot Syndrome Virus in genomes of decapod crustaceans

Andrey Rozenberg [*], Philipp Brand, Nicole Rivera, Florian Leese, Christoph D. Schubart

* Corresponding author: andrey.rozenberg@rub.de, jaera@yandex.com

Table S1 List of primers utilized for PCR

Scaffold	Position, bp	Region [a]	Forward primer	Reverse primer	Purpose [b]
I	5,408-6,008	TBP	GCAACACTGAGCCAGTTTAAG	TGTCATATTCACAGAAGGTTCC	S
I	21,109-21,713	Cytokine receptor	GCCGAGAATGCCCTTTGG	CGGTCTTTTTGCCGCTTTG	A
I	22,001-22,621	RR1	TCTCCCTAGTGGGGTGTCA	GCCTGGCAAAAGGGAGTG	R
I	35,356-35,516	C6-C7 boundary	TGGATGACTTTTACCAGCCT	GGAGAAGTGAGAGGTTGGC	S, R [c]
I	45,626-46,253	dUTPase	AGTGGATGAGCAGGGATCA	ATGCTGGTTGCAGCACCT	GB
I	54,992-55,578	wsv045	GCGTCCCGTTATTGGTGG	TGTTTGATTGCACAACCGTC	GB
I	61,399-61,914	wsv119	ACCGGATGCAACCGTTCTT	GCTCCTGAACCAGTTCGAC	S, GB
I	61,957~62,723	VP110 fragment	ACGGCCATCGCTGTTAGA	AAAACTCGTTTGGTCACATT	AC, GB
II	2,800-3,347	VP664:1	TTCTGGCAGGGCCATGAT	GGCCCTTGGCCTATGACT	Test
II	16,781-17,336	VP664:2	ACACCGATTATCTCAGACCT	TGAGGGACAGACTCTTGGTG	FS [e]
II	18,631-19,238	VP664:3	AGCGTCGGCGTCTTTACT	CCTCCCGCCATGTTGAGT	GB
II	23,720-24,289	wsv343:1	ACAGATTTGCCGGTTACACA	CAGAGGCGATGGTGCTCA	S
II	24,504-25,207	wsv343:2	AGATGCTGAACTATCCGAGT	TCTCGTCCTTGTCGAGAGA	FS, S
II	25,709-26,451	wsv343:3	TTGATGATGCCGACTACGA	ACCGGCGTGGTATCTCAT	GB, S
II	31,333-31,882	PK1	AACAACTTCCTCCGAAAACT	GAAGGGTGGTGGAGGTACT	GB
II	38,698-39,339	VP337	TCCAAACCTTGGACGCCT	GCCCCGGATTCTTCATCG	FS
II	42,247-42,847	VP90 fragment	CGTGTTCTGAGACATTGGTGA	TGACGCTATTTGTTTCGGCT	R

II	43,633-44,237	Endo fragment:1	CGTATGGAAGGCTATCGAGGA	CTCATGGCAGAAACCGGC	R
II	44,145-44,809	Endo fragment:2	GAGGGCGTCAGGTACAGG	GGCACACCCTTCTTGGGA	R [c]
III	274-588	C4 flank	GCCGCCAAATACCACTCC	CCAGACTTTCAATGTTTGTTGG	AC, A
III	17,006-17,190	C9 flank	TACTCTCCTCCGGTGGCT	GCAGGTCATCAGTTCTCTGC	GB
III	34,052-35,005	C9:1	CCTGTCAGGGTTATTGGGC	GAACACTGACACATATTTAGCC	LC [d]
III	35,643-36,839	C9:2	ACGCTGGTGACGATACCT	ACAACGTAGAAGGGAGAGAGA	LC [d]
III	42,621-42,929	VP136A	TGCCAAATTCGCTCGTGT	TCGAATGATGGTTCACTACAGA	S, R, FS [c]
III	44,779-45,539	VP53c	CGGATTAGATTCCCCACCC	CGCCTCGTGCTGAATTT	FS
III	52,321-52,982	Collagen	AGGACCGGTGTAGAAAGATGA	GGTGCAGCGAAAAAGCTG	GB
III	59,911-60,495	C10 flank	GTGAAAGAACCAGCGGGG	GCCTCCTTTCCTCCACGA	A
III	68,107-68,706	C1-C25 boundary	TCTACCGCCACCTCCTCT	TGTAGGTTCTTTTCGGGGT	S
III	73,043-73,539	VP39A	CGAATTCGTGTCTAGTCCCC	CAATGTCGCGACCGCC	GB
III	76,783-77,531	wsv161	AGCTGCATCCACCTGTACCTGCT	TCTAGCGTACCGCAGCGCGA	FS
III	78,242-79,116	DNApol:2	GCTTTATGGCTGTAAGAGCTCAACTCA	GCACGCCAAATCGGCCACTG	Test
III	79,685-80,493	DNApol:3	GACTGGCGGCCCCGGGTA	GGACGTGGAGACCACGGCAG	Test
III	83,263-84,022	DNApol:4	AACGATTTGGCCATGGCGTGC	CGTCTTCATCCCCGTCAAAAACCA	Test [e]
III	85,105-85,707	IAP:1	ACTGCAATGTAATTCTAGGCGG	CGTCAGATAGAGGGAAACCCA	Test
III	85,952-86,560	IAP:2	TTTAGAACCGCCATCATCT	AACAGGCAATGTATTAAACGA	GB
III	87,743-88,368	wsv293a	AGCGGAGAAAGGCGAGAA	CCTCGGTTAAGCATGCGG	GB

[a] regions are named according to respective proteins in WSSV, or their ORF number (prefixed with "wsv"), or *Metopaulias* contig number (prefixed with "C")
[b] A – ambiguous positions, AC – anomalous coverage, FS – suspected frame shift, GB – confirmation of ORF boundaries, LC – low coverage, R – repetitive region, S – scaffolding, Test – test fragments
[c] considerably truncated Sanger-sequences obtained due to secondary products or polymorphisms
[d] no PCR product
[e] primer pairs used for the PCR-assay

Table S2 Results of blastp searches for the predicted protein sequences from the viral scaffolds of *Metopaulias depressus* against GenBank

Scaf-fold	Position, bp	Predicted protein length	WSSV-CH homolog	Best blastp hits						
				Accession number	E-value	Description	Organism	Pairwise identity, %	Query coverage, %	Alignment length
I	2,517-3,356	279	wsv299	NP_477821	6.1e-06	wsv299	WSSV-CH	30.4	38.6	115
I	3,524-5,980	818	wsv303	NP_477825	0	wsv303	WSSV-CH	48.8	98.8	883
I	8,084-12,361	1,425	wsv151	AFX59529	1.7e-82	wsv151	WSSV-CH	28.0	58.1	917
I	13,933-17,373	1,145	wsv011	AFX59388	0	wsv011	WSSV-CH	44.9	99.5	1,202
I	19,141-21,198	685	wsv220	AAL89143	1.0e-141	WSSV275	WSSV-TW	37.9	99.4	700
I	22,400-24,835	811	wsv172	NP_477694	0	wsv172	WSSV-CH	55.5	99.5	840
I	24,958-28,317	1,119	wsv166	NP_477688	0	wsv166	WSSV-CH	37.2	92.0	1,078
I	29,688-35,294	1,868	wsv447	AAL89375	0	WSSV507	WSSV-TW	45.1	77.4	1,476
I	36,408-43,088	2,227 [a]	wsv143	AAK77753	1.2e-112	ORF84	WSSV-TH	31.0	43.9	1,058
I	43,108-44,328	406	wsv188	XP_005953175	2.1e-144	Ribonucleoside-diphosphate reductase subunit M2-like isoform X1	*Haplochromis burtoni*	58.5	93.9	390
I	45,135-45,998	288	dUTPase no in WSSV	XP_002973182	8.7e-38	hypothetical protein SELMODRAFT_232059	*Selaginella moellendorffii*	51.0	53.8	155
I	46,200-47,384	394	wsv446	NP_477968	5.4e-16	wsv446	WSSV-CH	28.7	55.4	230
I	47,874-52,274	1,466	wsv465	ACK58409	7.2e-17	ORF16	WSSV-TH	24.0	30.8	483
I	52,821-55,352	843	wsv045	NP_477567	1.7e-167	wsv045	WSSV-CH	39.0	79.6	782
I	55,376-58,807	1,143	wsv037	AAK77711	1.9e-162	ORF42	WSSV-TH	54.1	41.4	497
I	59,002-61,614	870	wsv119	NP_477641	0	wsv119	WSSV-CH	43.4	74.7	671
I	61,759-62,034	91	wsv035	NP_477557	5.2e-20	wsv035	WSSV-CH	51.6	96.7	91
II	898-18,972	6,024	wsv360	AFX59737	0	wsv360	WSSV-CH	50.9	99.2	6,185
II	19,204-31,302	4,031	wsv343			no hits				
II	31,716-34,289	857	wsv423	AAL30163	0	protein kinase 1	WSSV-TW	45.4	71.8	635
II	34,316-34,906	196	wsv421	NP_477833	2.2e-05	wsv311	WSSV-CH	35.4	46.7	99
II	35,421-38,787	1,120 [a]	wsv139	NP_477661	0	wsv139	WSSV-CH	41.7	70.2	856
II	38,756-39,691	311	wsv137	NP_477659	6.3e-25	wsv137	WSSV-CH	28.5	98.7	319

II	40,142-41,488	448 [a]	wsv131	NP_477653	2.2e-71	wsv131	WSSV-CH	34.0	96.4	470
II	41,511-42,371	286	wsv133	NP_477655	4.6e-40	wsv133	WSSV-CH	39.1	83.6	256
III	561-848	95	wsv021			no hits				
III	927-1,754	275	wsv023	AAL88947	8.1e-62	WSSV079	WSSV-TW	39.8	93.5	279
III	1,820-2,215	131	wsv025	AFX59402	3.3e-02	wsv025	WSSV-CH	51.5	25.0	33
III	2,231-6,865	1,543	wsv026	NP_477548	0	wsv026	WSSV-CH	50.3	98.7	1,572
III	8,195-11,185	996	wsv226	AFX59603	5.2e-108	ORF114	WSSV-TH	31.3	80.0	825
III	11,727-14,753	1,008	wsv192	NP_477714	0	wsv192	WSSV-CH	45.3	98.3	1,036
III	14,831-15,739	302	wsv191	AAW88443	1.8e-80	non-specific nuclease	WSSV-CH	47.2	95.1	303
III	17,211-18,929	572	wsv427	NP_477949	7.7e-117	wsv427	WSSV-CH	37.2	96.0	616
III	19,167-22,964	1,265	wsv433	NP_477955	0	wsv433	WSSV-CH	51.2	99.7	1,321
III	23,005-24,687	560	wsv440	AAK77674	2.8e-51	ORF5	WSSV-TH	54.5	31.0	176
III	24,642-27,239	865	wsv442	AFX59819	1.8e-117	wsv442	WSSV-CH	30.8	99.9	896
III	30,327-33,310	994 [a]	wsv064+063	EGZ20325	1.3e-08	hypothetical protein PHYSODRAFT_406923	*Phytophthora sojae*	35.8	10.7	109
III	38,213-40,172	653	wsv277	NP_477799	9.3e-105	wsv277	WSSV-CH	35.1	87.6	647
III	40,595-44,461	1,289	wsv271	NP_477793	3.4e-85	wsv271	WSSV-CH	42.9	32.4	431
III	44,426-44,971	181	wsv270	NP_477792	3.9e-25	wsv270	WSSV-CH	38.4	82.4	159
III	44,938-46,377	479	wsv269	NP_477791	0	wsv269	WSSV-CH	55.7	97.9	483
III	46,469-47,209	246	wsv267	NP_477789	1.7e-26	wsv267	WSSV-CH	36.5	68.8	192
III	47,325-49,637	770	wsv260	AAL89183	5.4e-37	WSSV315	WSSV-TW	29.8	48.9	406
III	50,920-52,581	553	wsv001	NP_477523	7.0e-21	wsv001	WSSV-CH	25.5	74.7	478
III	52,725-55115	796	wsv332	NP_477854	0	wsv332	WSSV-CH	39.2	99.0	802
III	55,224-57,680	818	wsv327	NP_477849	0	wsv327	WSSV-CH	42.8	95.2	800
III	58,441-59,865	473	wsv147	NP_477669	6.9e-05	wsv147	WSSV-CH	22.6	74.5	376
III	62,523-63,143	206	wsv419	NP_477941	1.8e-21	wsv419	WSSV-CH	34.2	93.2	193
III	62,963-64,516	517	wsv415	AFX59792	1.4e-139	wsv415	WSSV-CH	42.8	99.8	547
III	64,652-65,005	117	wsv414	AAL89341	7.5e-13	WSSV473	WSSV-TW	38.3	98.3	120
III	65,332-65,604	90	wsv324	NP_477846	9.8e-05	wsv324	WSSV-CH	33.7	89.0	89

III	65,906-66,142	78	wsv322	NP_477844	1.3e-03	wsv322	WSSV-CH	32.4	87.3	71
III	66,363-66,752	129	wsv321	AFX59698	7.8e-21	wsv321	WSSV-CH	47.0	76.9	100
III	66,908-69,883	991	wsv313	AFX59690	3.0e-113	wsv313	WSSV-CH	40.2	49.8	569
III	69,974-70,567	197	wsv311	NP_477833	1.6e-08	wsv311	WSSV-CH	33.3	77.8	156
III	70,645-71,418	257	wsv310	NP_477832	1.6e-39	wsv310	WSSV-CH	38.0	83.7	216
III	71,415-72,818	467	wsv308	AAK77820	1.6e-124	ORF151	WSSV-TH	44.8	98.1	471
III	72,809-73,111	100	wsv306	NP_477828	3.3e-23	wsv306	WSSV-CH	65.7	66.3	67
III	73,422-75,302	626	wsv282	NP_477804	0	wsv282	WSSV-CH	60.4	78.3	513
III	75,443-77,426	660 [a]	wsv161	AFX59539	2.3e-73	wsv161	WSSV-CH	46.2	44.8	303
III	77,503-84,399	2,298	wsv514	AFX59875	0	putative DNA polymerase	WSSV from S Korea	55.1	99.9	2,392
III	84,915-86,372	485	IAP not in WSSV	AHC55308	2.3e-52	inhibitor of apoptosis protein	*Palaemon carinicauda*	35.2	60.3	347
III	86,662-87,348	228	wsv295	AAL89219	9.3e-03	WSSV351	WSSV-TW	37.5	21.0	48
III	87,428-87,922	164	wsv294	no hits						
III	88,010-88,258	82	wsv293a	YP_007003111	4.3e-21	wsv293a	WSSV-CH	65.2	79.5	66
III	88,215-92,825	1,536	wsv289	AAK77812	0	ORF143	WSSV-TH	38.5	97.7	1,581
III	93,236-96,400	1,054	wsv285	NP_477807	2.0e-132	wsv285	WSSV-CH	43.8	49.0	536
III	96,852-98,046	398 [a]	wsv078	NP_477600	1.4e-01	wsv078	WSSV-CH	29.0	21.6	93

[a] based on protein sequences after "correction" of the frameshifts

Table S3 ORFs and ORF fragments with homology to WSSV, dUTPases or Inhibitor of Apoptosis Proteins (IAPs) in the sequences of repetitive families identified in the *Penaeus monodon* fosmid library (Huang et al, 2011)

Repetitive element family	Position, bp	Strand	WSSV-CH homolog [a]	Predicted protein length, aa	Truncated ends	Interruptions	≥3 in-frame Ns in row	Orthologue presence in *Metopaulias*
FAM1	6,489-7,926	+	wsv269	~478		Frameshift		+
FAM1	13,803-15,212	-	wsv308*	469				+
FAM1	15,221-16,342	-	wsv306	373				+
FAM2	1-18,284	+	wsv360	~6,093		Frameshift	+	+
FAM5	1-1,825	-	wsv440	608				+
FAM5	1,956-4,973	-	wsv433	1,005	Truncated 5'-end			+
FAM5	6,465-8,234	+	wsv427	589				+
FAM6	71-721	-	wsv021*	216				+
FAM6	794-1,732	-	wsv023*	312				+
FAM9 15-44	75-2,390	+	wsv442*	771			+	+
FAM9 15-44	5,315-7,558	-	wsv220	747				+
FAM9 15-44	7,489-10,920	+	wsv216	1,143		Stop-codon	+	-
FAM9 15-44	11,506-14,109	-	wsv119	867				+
FAM9 15-44	14,180-17,38	-	wsv115	952		Stop-codon		-
FAM9 15-44	17,205-19,55	-	wsv131*	616			+	+
FAM9 15-44	19,444-20,205	+	wsv134	253				-
FAM9 15-44	20,700-23,792	-	wsv143*	1,030	3'-fragment [b]			+
FAM24	1,5-1,334	-	wsv136*	109				-
FAM24	1,495-4,491	-	wsv192	998				+
FAM24	4,866-9,464	+	wsv209	1,532				-
FAM28	1,312-2,772	+	wsv325	486				-
FAM28	2,987-6,481	-	wsv271	1,164			+	+
FAM29	81-2,426	+	wsv423	781				+
FAM31&207	143-3,79	+	wsv313*	978				+
FAM31&207	5,165-6,679	+	IAP not in WSSV	504				(+)
FAM31&207	9,967-10,560	+	wsv206*	197				-
FAM31&207	11,763-24,78	+	wsv343	~4,104		Frameshift	+	+
FAM43	1-4,347	-	wsv151*	1,449				+

FAM43	4,376-7,919	+	wsv011	1,181			+	+
FAM46	673-1,587	+	IAP<br>not in WSSV	304			+	(+)
FAM56	2,669-4,240	-	wsv115	523	3'-fragment [b]			-
FAM57	672-2,867	+	wsv220*	731			+	+
FAM87	442-7,128	+	wsv514	2,228			+	+
FAM87	7,373-10,615	+	wsv226*	1,080				+
FAM124	565-4,923	-	wsv026	1,452				+
FAM137	1-2,547	-	wsv303	849				+
FAM138	1-2,19	-	wsv433	673	5'-fragment [b]		+	+
FAM139	90-2,431	+	wsv360	780	Middle-fragment [b]		+	+
FAM142	1,739-2,598	+	wsv037*	286	5'-fragment [b]			+
FAM145	276-1,646	+	wsv423*	456	Truncated 5'-end		+	+
FAM145	1,985-2,485	+	dUTPase<br>not in WSSV	166				(+)
FAM145	2,608-3,792	+	wsv440*	394	5'-fragment [b]		+	+
FAM146	115-2,538	-	wsv216*	807	Truncated 5'-end			-
FAM152	1-423	+	wsv139*	140	3'-fragment			+
FAM152	402-1,646	+	wsv137*	414			+	+
FAM152	3,836-9,247	-	wsv447	1,803			+	+
FAM152	9,383-11,809	-	wsv332	808				+
FAM152	11,886-14,417	+	wsv327	843				+
FAM152	14,617-15,984	+	wsv282	455	Truncated 5'-end			+
FAM152	17,262-19,748	+	wsv285	828			+	+
FAM152	21,997-22,794	+	wsv244*	265	3'-fragment			-
FAM156 3,4	1-1,752	-	wsv139	584	Middle fragment		+	+
FAM156 1,2,5	954-2,180	+	wsv161*	409	5'-fragment [b]			+
FAM158	63-317	+	wsv293a*	84				+
FAM158	389-3,912	+	wsv289	1,174	Truncated 3'-end [b]			+
FAM177	108-2,24	+	wsv433	638	3'-fragment		+	+
FAM179	1-1,815	-	wsv037	605	5'-fragment [b]			+
FAM179	1,904-4,722	+	wsv035	~938		Frameshift		+
FAM197	1,808-3,181	-	wsv289	457	3'-fragment [b]			+
FAM199	182-1,660	+	wsv415*	492			+	+

FAM209	29-2,821	+	wsv035	930			+	+
FAM224&1875	146-2,462	+	wsv360	772	Middle-fragment		+	+
FAM245	20-1,496	-	wsv045*	~491		Frameshift		+
FAM255	994-1,720	-	wsv447*	242	5'-fragment		+	+
FAM255	1,977-8,153	+	wsv514	~2,058	Truncated 5'-end [b]	Frameshift	+	+
FAM259	1,1-2,281	-	wsv306	426				+
FAM259	2,369-4,930	-	wsv332	853			+	+
FAM278	676-2,232	-	wsv313*	518	3'-fragment		+	+
FAM327	1,81-3,714	-	wsv139*	877	3'-end truncated		+	+
FAM328	288-2,576	-	wsv199*	762				-
FAM346	1-1,57	-	wsv011	352	3'-fragment [b]			+
FAM361	712-4,948	-	wsv209	~1,411		Frameshift	+	-
FAM411	1-1,739	+	wsv037	579	3'-fragment [b]	Frameshift		+
FAM472	1-1,850	-	wsv447	642	3'-fragment [b]			+
FAM483	2,74-3,624	-	wsv360	516	Fragment [b]			+
FAM541	24-3,175	+	wsv343	1,050	Middle-fragment [b]		+	+
FAM574	1-3,690	-	wsv026	1,230			+	+
FAM838	1-1,616	+	wsv360	538	Middle-fragment [b]		+	+
FAM1203	1-1,179	-	wsv447*	393	Middle fragment			+

[a] homologs not reported in the original study (Huang et al, 2011) are marked with asterisk
[b] ORF truncations associated with ends of the respective repetitive element family sequence

Table S4 ORFs and ORF fragments with homology to WSSV, dUTPases or Inhibitor of Apoptosis Proteins (IAPs) found in the sequence of the Mj024A04 BAC-clone from *Penaeus japonicus* (Koyama et al, 2010)

Piece	Gene number [a]	Position, bp	Strand	WSSV-CH homolog	Predicted protein length, aa	Comments	Orthologue presence in *Metopaulias*
1	01	1,819-3,459	+	IAP	546		(+)
1	06A	20,293-22,593	+	IAP	766	4 exons	(+)
1	06B	23,123-25,180	+	IAP	685		(+)
3	11	8,733-12,965	+	wsv209	1,410		-
3	13	17,33-21,862	+	wsv289	1,609		+
3	14	21,971-34,834	-	wsv343	4,287		+
3	15	34,973-37,9	-	wsv327	678		+
3	16	37,164-39,560	+	wsv332	798		+
3	17	39,637-40,959	+	wsv306	440		+
3	18	45,702-48,470	+	wsv285	922		+
3	22	53,906-54,346	-	dUTPase	146		(+)
3	24	63,670-64,680	-	IAP	336		(+)

[a] gene designation according to Koyama et al, 2010
