# Supplementary material for: Characterization of fossilized relatives of the White Spot Syndrome Virus in genomes of decapod crustaceans
Source: BMC Evol Biol. 2015 Jul 19;15:142. doi: 10.1186/s12862-015-0380-7 (PMC4506587; doi:10.1186/s12862-015-0380-7)
Supplement: Additional file 2: Figure S1. — Agarose gel electrophoresis of a diagnostic PCR-product for a subset of Metopaulias individuals. The product corresponds to a fragment of the wsv360 homolog (primer pair “VP664:2”). The first and the last lanes are size-standards. The penultimate lane is a negative control. Figure S2. Polymorphic position on the sequencing chromatograms for a PCR product from the homolog of DNA polymerase. Shown are 17 bases around the A/G silent polymorphism in the fragment amplified with primers VP664:2. Among the 5 individuals represented the bottommost one is the individual, for which the original 454-library was prepared. Figure S3. Full ML phylogenetic tree for DNA polymerase amino acid sequences. Figure S4. ML phylogenetic tree for helicase amino acid sequences (alignment length 624 residues). The tree is formally rooted on the split between Bacteria and Eukaryota. Figure S5. Full ML phylogenetic tree for ribonucleotide reductase large subunit amino acid sequences. Figure S6. ML phylogenetic tree for ribonucleotide reductase small subunit amino acid sequences (alignment length 316 residues). The tree is rooted on the split between Bacteria and Eukaryota. Figure S7. Full ML phylogenetic tree for TATA-box binding protein (TBP) amino acid sequences. Figure S8. Full ML phylogenetic tree for endonuclease amino acid sequences. Figure S9. ML phylogenetic tree for protein kinase amino acid sequences (alignment length 227 residues). The tree is formally rooted by the sequences from the main part of Bacteria. Figure S10. Full ML phylogenetic tree for BIR-domains of Inhibitor of Apoptosis Proteins. The tree is formally rooted by a clade of predominantly vertebrate sequences. Figure S11. ML phylogenetic tree for RING-domains of Inhibitor of Apoptosis Proteins (alignment length 53 residues). The tree is formally rooted by a clade of predominantly vertebrate sequences. Figure S12. ML phylogenetic tree for dUTPase amino acid sequences (alignment length 137 residues). The tree is rooted on t [file 12862_2015_380_MOESM2_ESM.pdf]

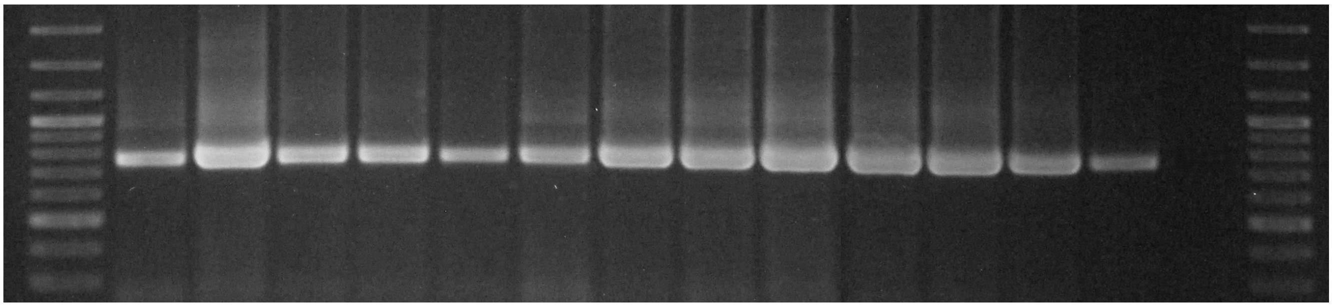

Figure S1. Agarose gel electrophoresis of a diagnostic PCR-product for a subset of Metopaulias individuals. The product corresponds to a fragment of the wsv360 homolog (primer pair “VP664:2”). The first and the last lanes are size-standards. The penultimate lane is a negative control.

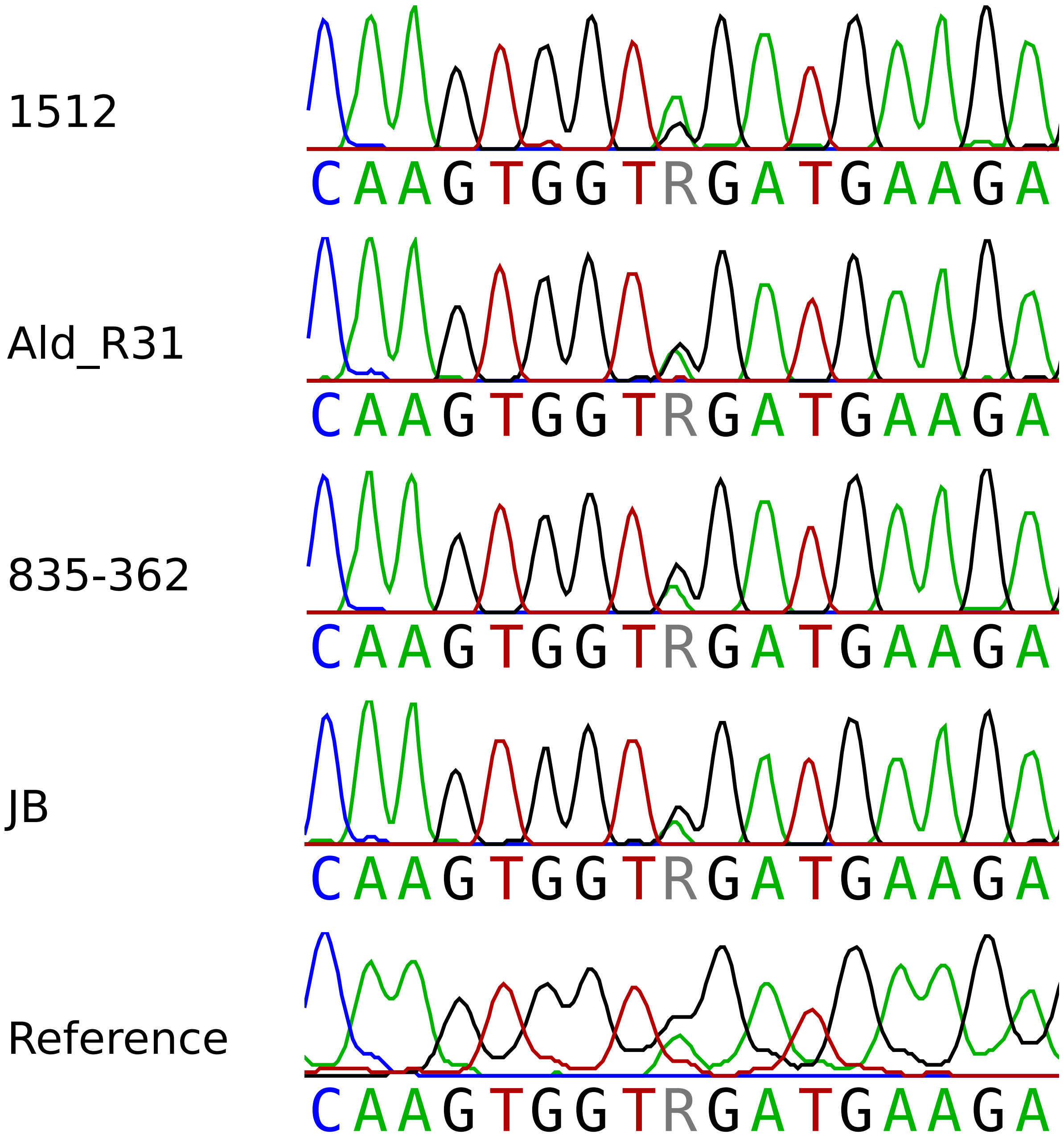

Figure S2. Polymorphic position on the sequencing chromatograms for a PCR product from the homolog of DNA-polymerase. Shown are 17 bases around the A/G silent polymorphism in the fragment amplified with primers VP664:2. Among the 5 individuals represented the bottommost one is the individual, for which the original 454-library was prepared.

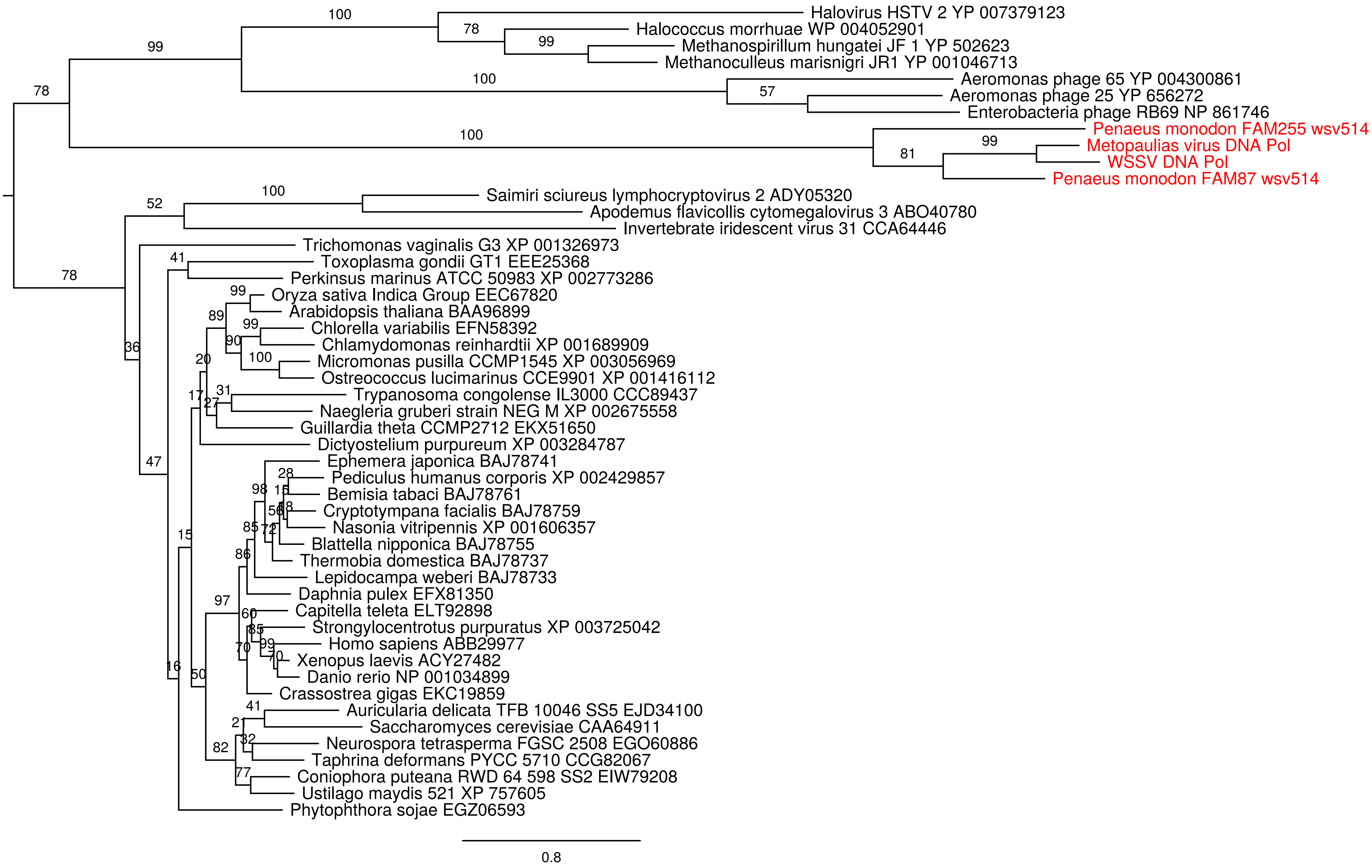

Figure S3. Full ML phylogenetic tree for DNA-polymerase amino-acid sequences.

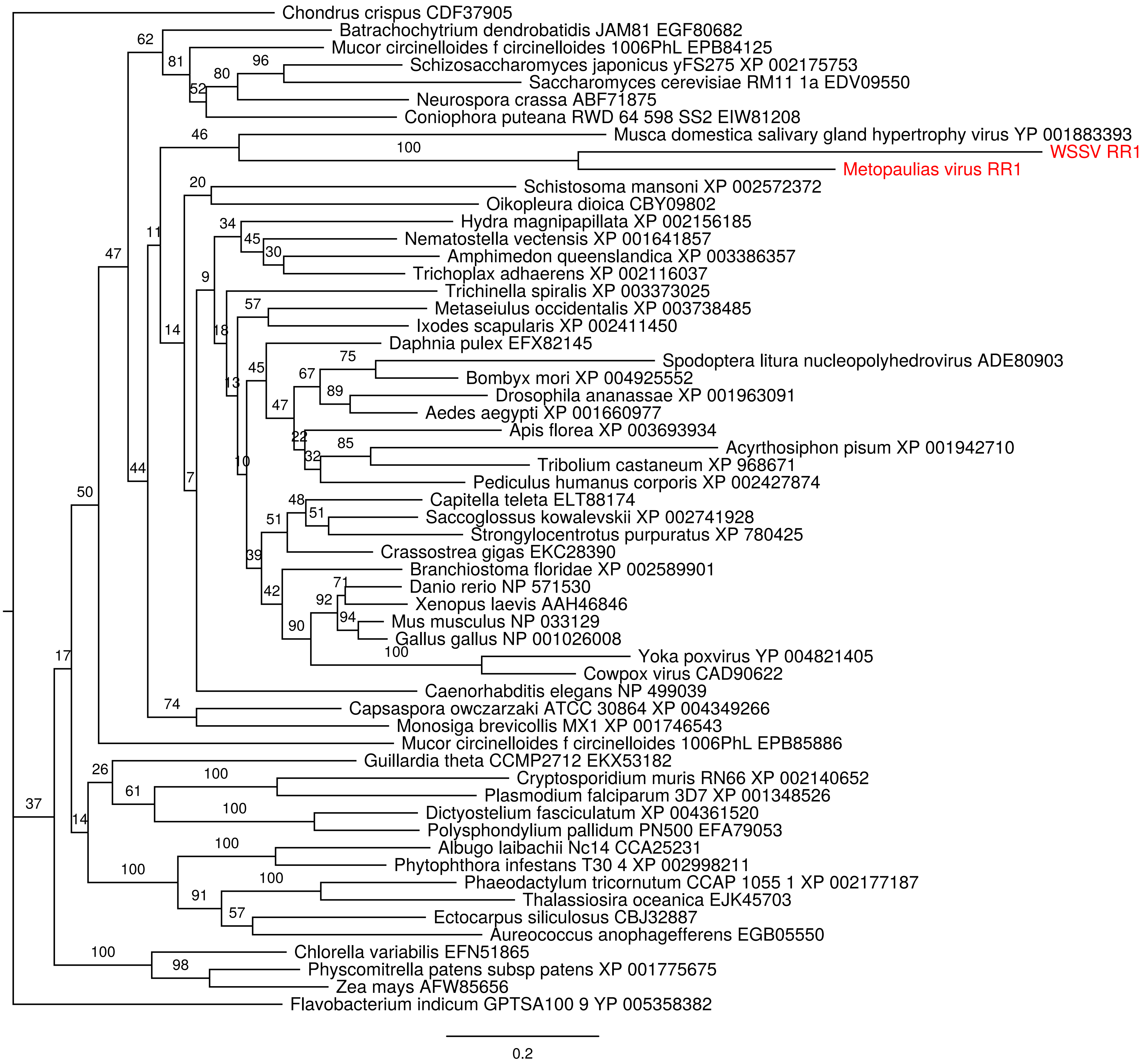

Figure S4. Full ML phylogenetic tree for ribonucleotide reductase large subunit amino-acid sequences.

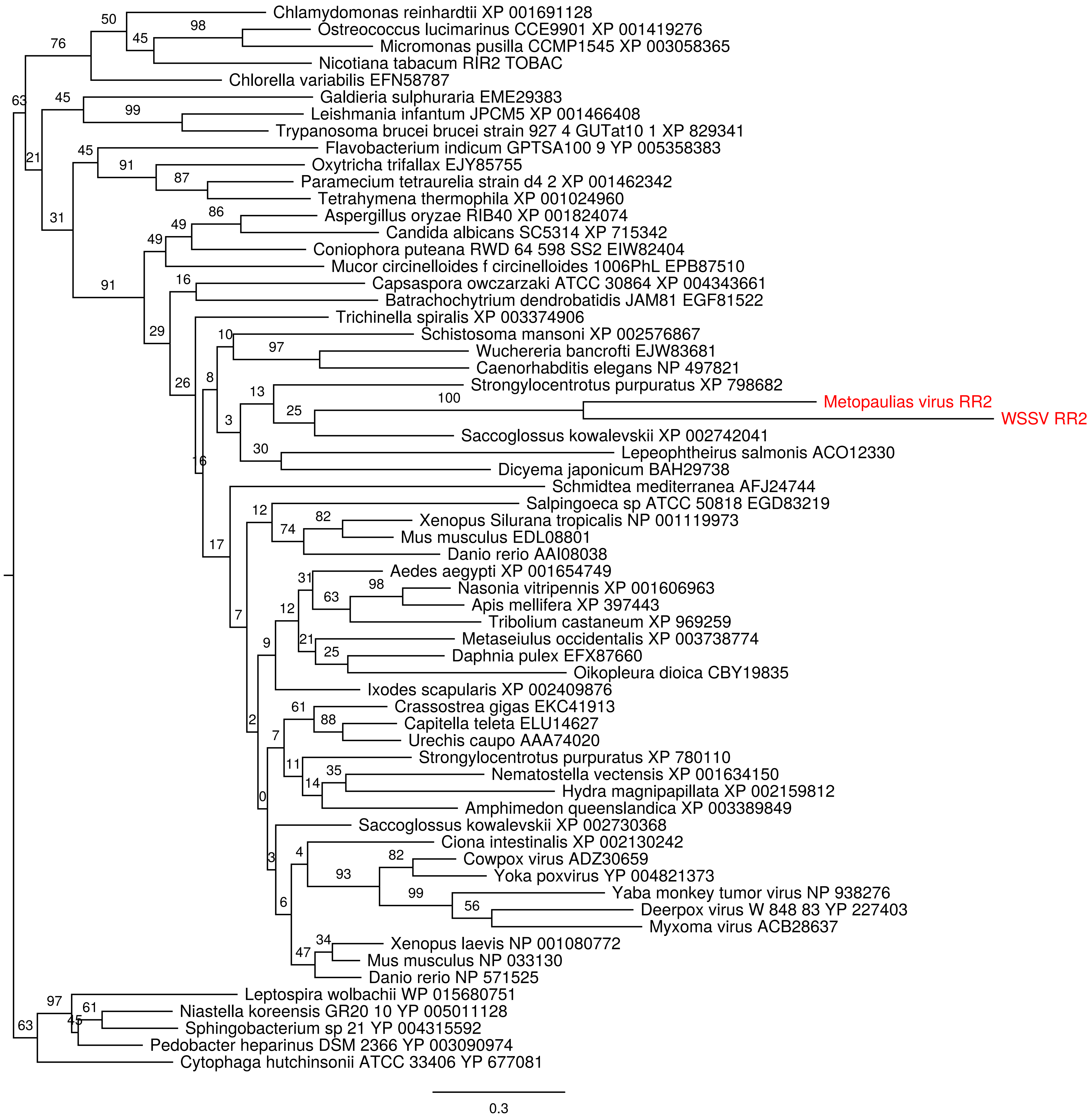

Figure S5. ML phylogenetic tree for ribonucleotide reductase small subunit amino-acid sequences (alignment length 316 residues). The tree is rooted on the split between Bacteria and Eukaryota.

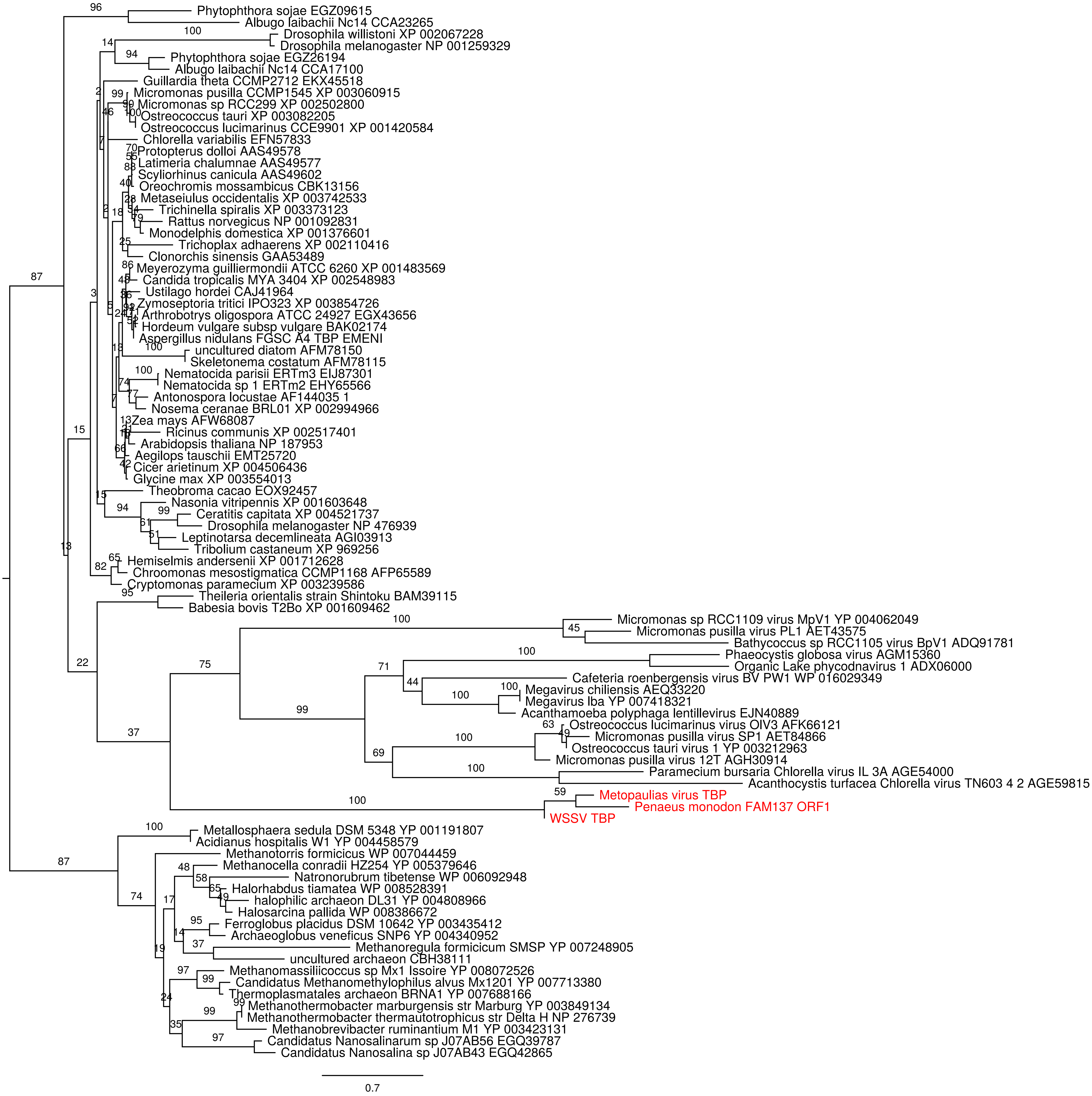

Figure S6. Full ML phylogenetic tree for TATA-box binding protein (TBP) amino-acid sequences.

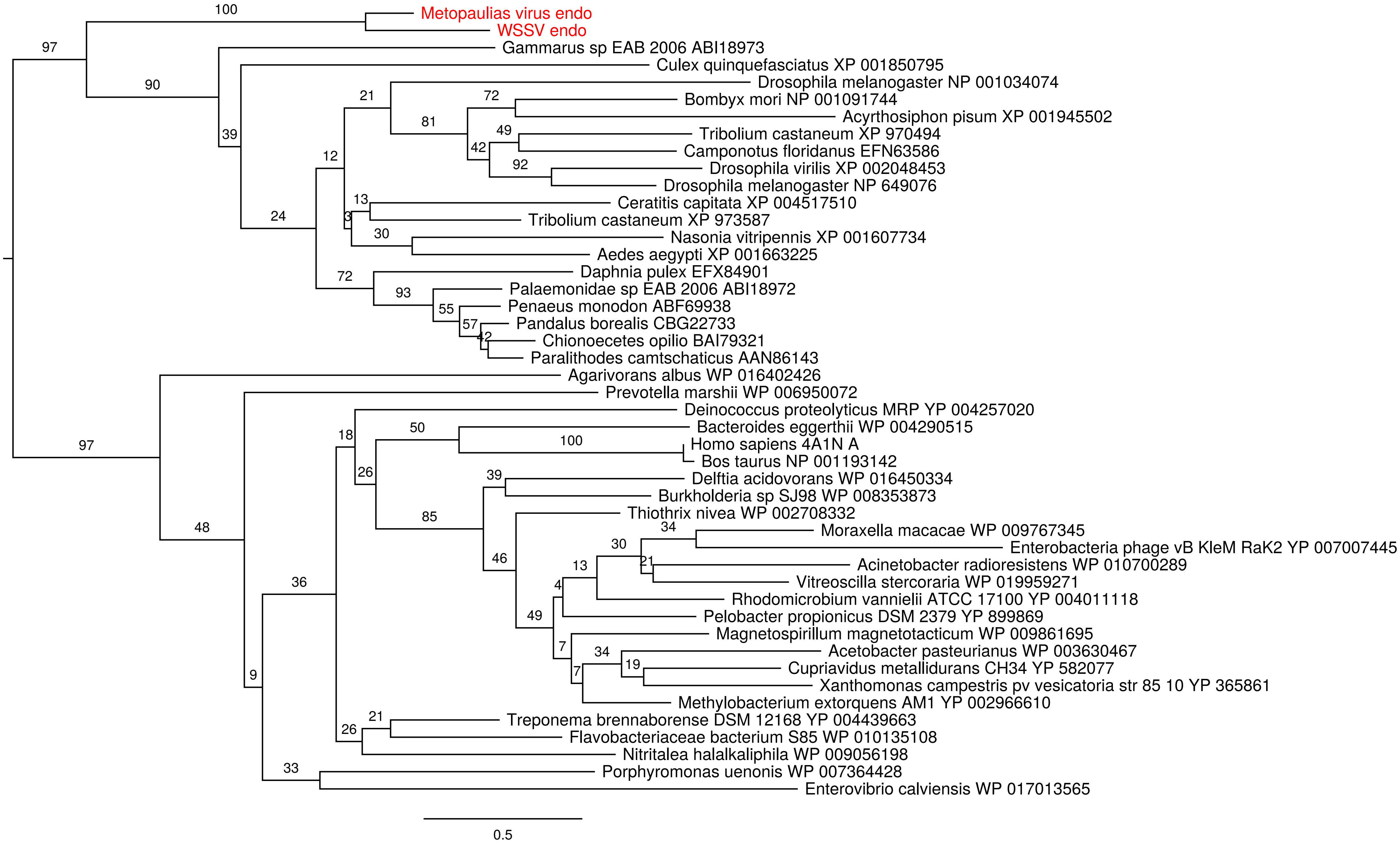

Figure S7. Full ML phylogenetic tree for endonuclease amino-acid sequences.

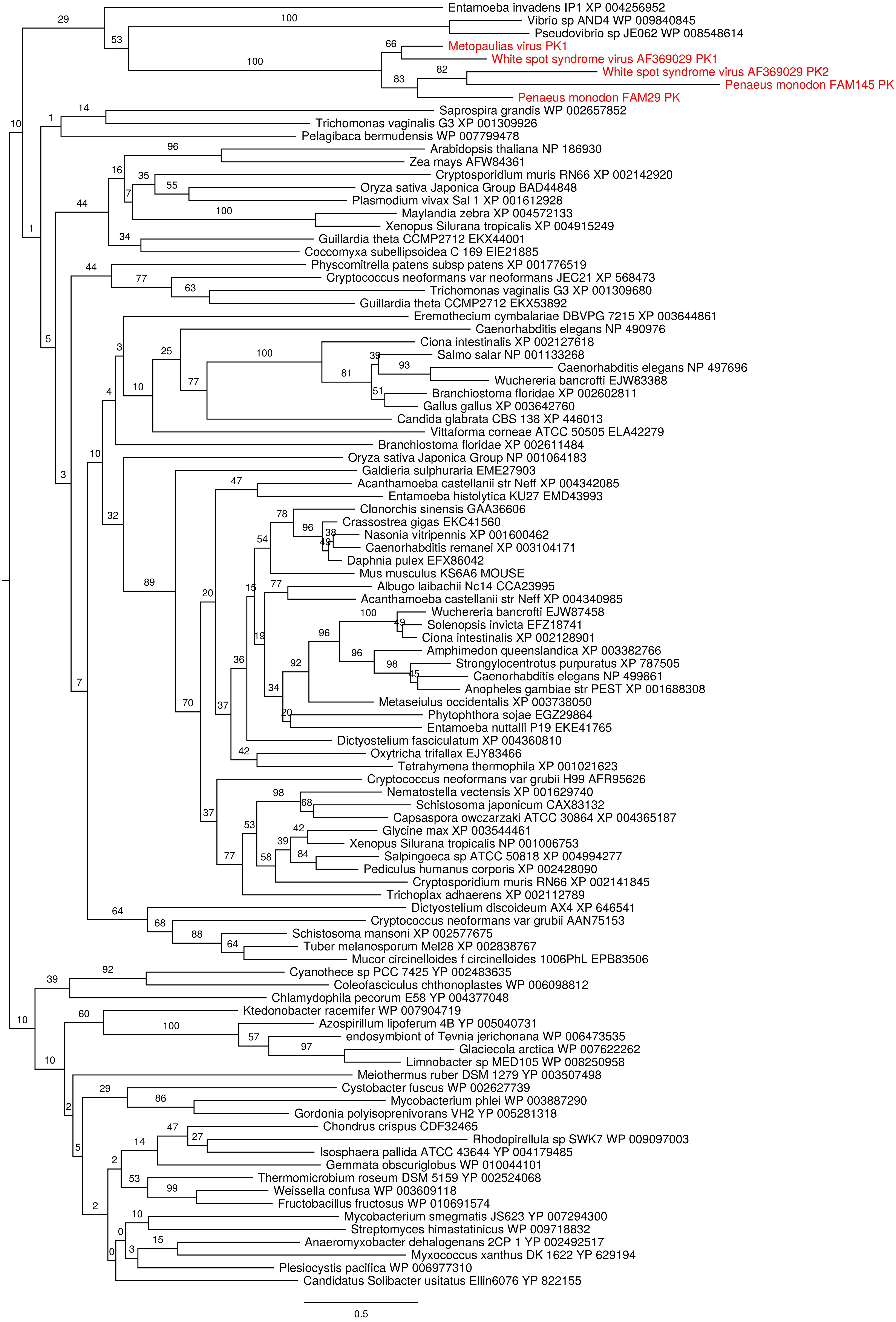

Figure S8. ML phylogenetic tree for protein kinase amino-acid sequences (alignment length 227 residues). The tree is formally rooted by the sequences from the main part of Bacteria.

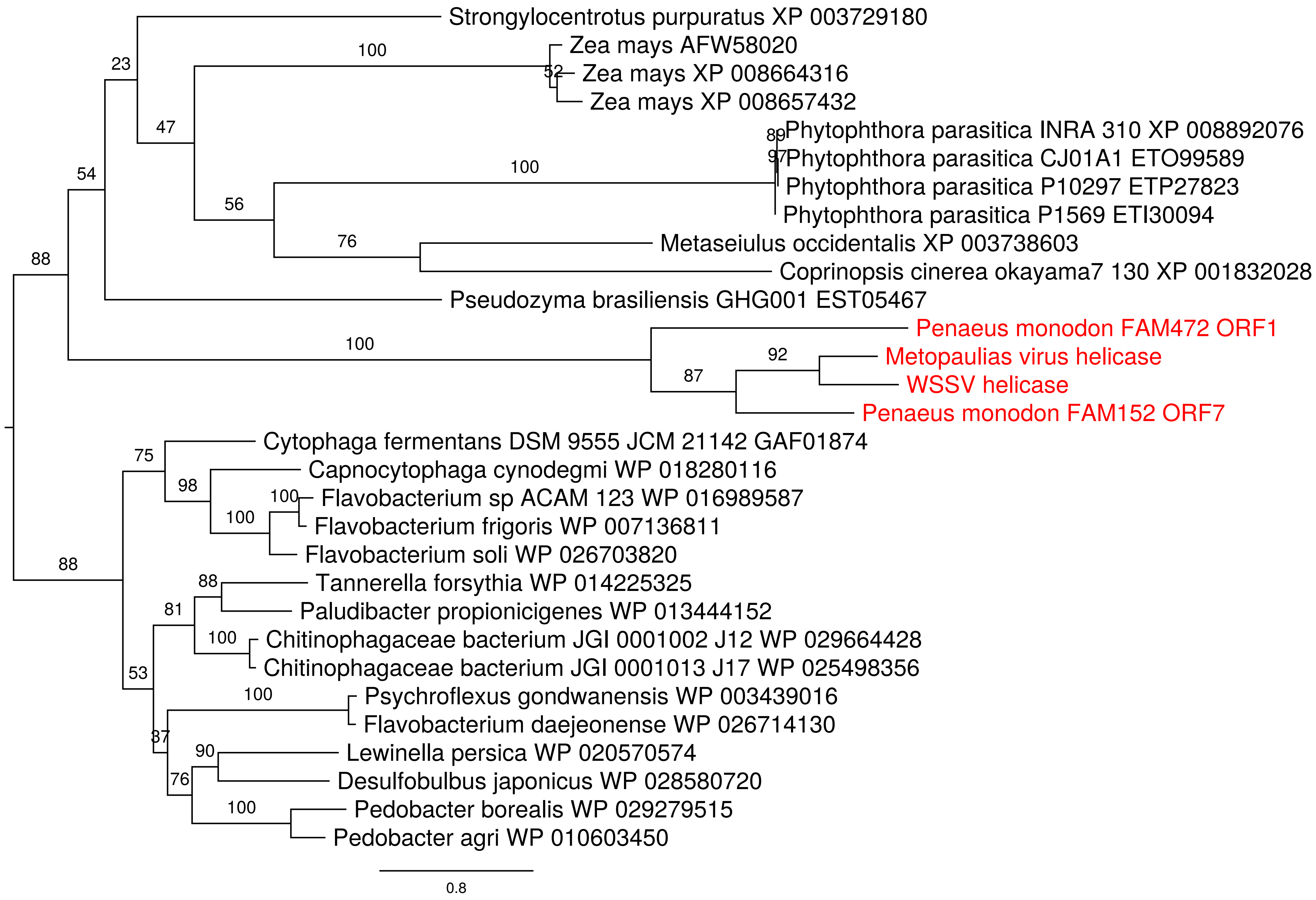

Figure S9. ML phylogenetic tree for helicase amino-acid sequences (alignment length 624 residues). The tree is formally rooted on the split between Bacteria and Eukaryota.

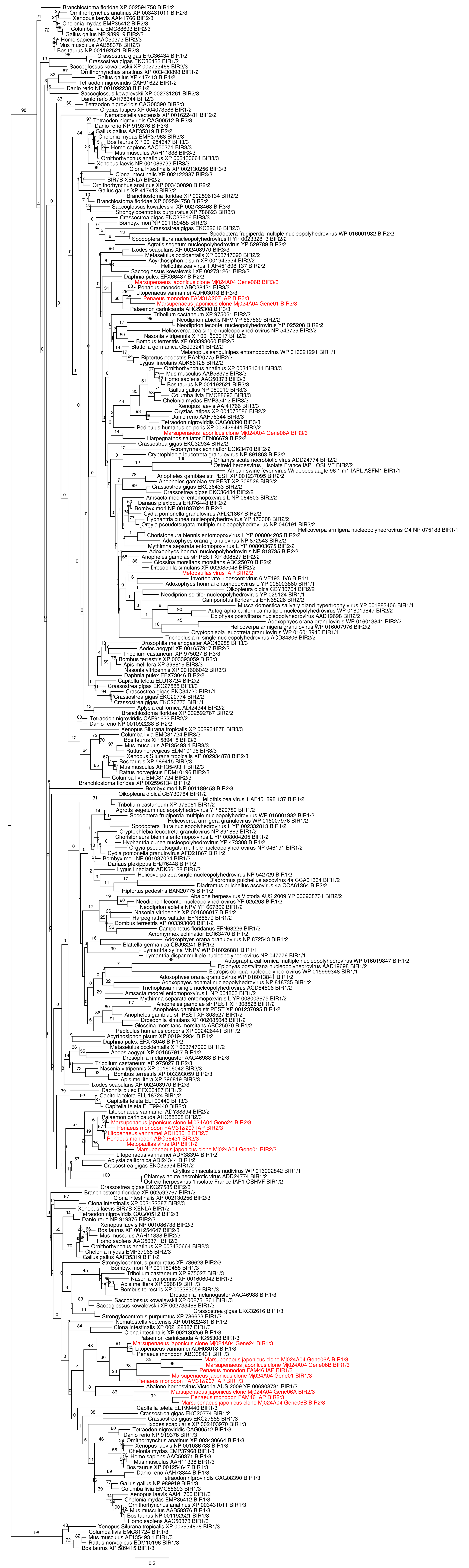

Figure S10. Full ML phylogenetic tree for BIR-domains of Inhibitor of Apoptosis Proteins. The tree is formally rooted by a clade of predominantly vertebrate sequences.

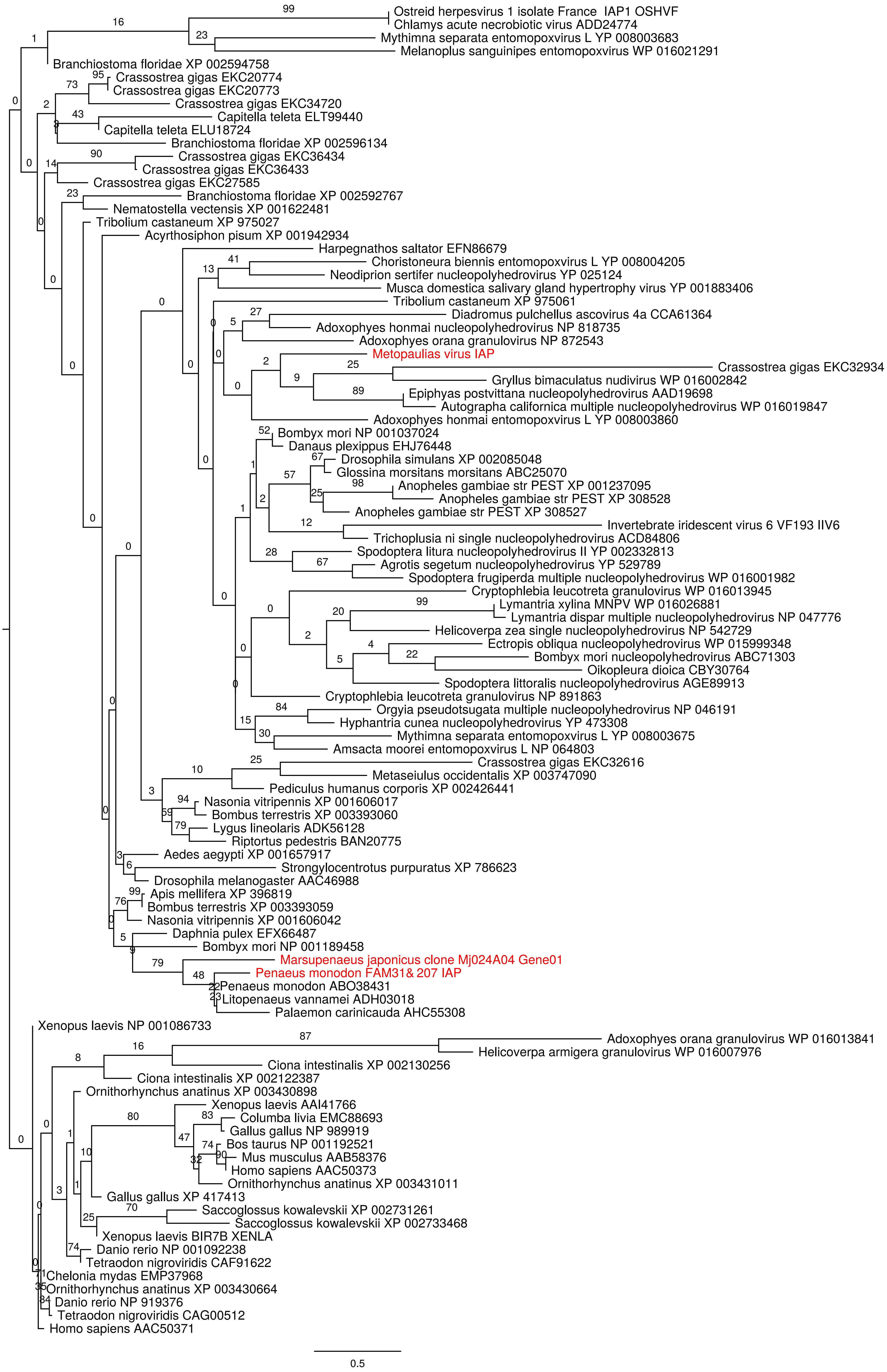

Figure S11. ML phylogenetic tree for RING-domains of Inhibitor of Apoptosis Proteins (alignment length 53 residues). The tree is formally rooted by a clade of predominantly vertebrate sequences.

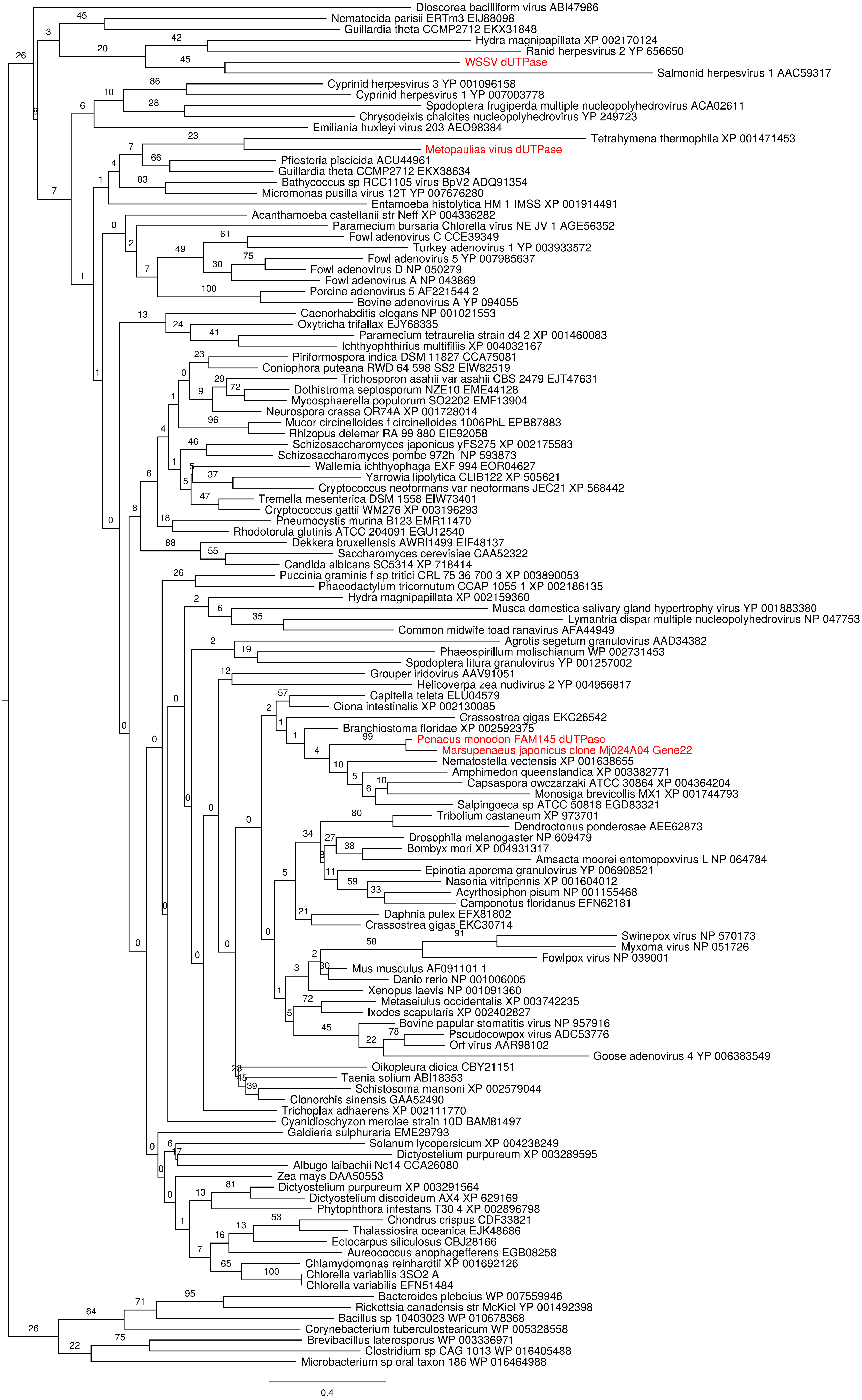

Figure S12. ML phylogenetic tree for dUTPase amino-acid sequences (alignment length 137 residues). The tree is rooted on the split between Bacteria and Eukaryota.
